# The *Drosophila* protein, Nausicaa, regulates lamellipodial actin dynamics in a Cortactin-dependent manner

**DOI:** 10.1242/bio.038232

**Published:** 2019-06-04

**Authors:** Meghan E. O'Connell, Divya Sridharan, Tristan Driscoll, Ipsita Krishnamurthy, Wick G. Perry, Derek A. Applewhite

**Affiliations:** 1 Department of Biology, Reed College, Portland, Oregon 97202, USA; 2 Department of Molecular Genetics and Cell Biology, University of Chicago, Chicago, Illinois 60637, USA; 3 Department of Biology, Boston College, Chestnut Hill, Massachusetts 02467, USA; 4 Department of Cardiovascular Medicine, Yale University, New Haven, Connecticut 06511, USA

**Keywords:** Actin filament dynamics, Cell migration, Cortactin, Cortactin binding proteins

## Abstract

*Drosophila* CG10915 is an uncharacterized protein coding gene with sequence similarity to human Cortactin-binding protein 2 (CTTNBP2) and Cortactin-binding protein 2 N-terminal-like (CTTNBP2NL). Here, we have named this gene *Nausicaa* (*naus*) and characterize it through a combination of quantitative live-cell total internal reflection fluorescence microscopy, electron microscopy, RNAi depletion and genetics. We found that Naus co-localizes with F-actin and Cortactin in the lamellipodia of *Drosophila* S2R+ and D25c2 cells and this localization is lost following Cortactin or Arp2/3 depletion or by mutations that disrupt a conserved proline patch found in its mammalian homologs. Using permeabilization activated reduction in fluorescence and fluorescence recovery after photobleaching, we find that depletion of Cortactin alters Naus dynamics leading to a decrease in its half-life. Furthermore, we discovered that Naus depletion in S2R+ cells led to a decrease in actin retrograde flow and a lamellipodia characterized by long, unbranched filaments. We demonstrate that these alterations to the dynamics and underlying actin architecture also affect D25c2 cell migration and decrease arborization in *Drosophila* neurons. We present the hypothesis that Naus functions to slow Cortactin's disassociation from Arp2/3 nucleated branch junctions, thereby increasing both branch nucleation and junction stability.

## Abstract (precis)

**Summary:** The *Drosophila* Cortactin-binding protein Nausicaa regulates actin branching and lamellipodial dynamics through is anchoring of Cortactin in the lamellipodia.

## INTRODUCTION

Cell migration is critical to a number of physiological processes including wound healing and immune function, development, neurogenesis and vascularization. Aberrant cell migration leads to a number of diseases including schizophrenia and mental disabilities, immunodeficiency, craniofacial disorders and metastasis (Ridley et al., 2003). Cell migration relies heavily on the actin cytoskeleton, and proceeds in four major steps - protrusion, adhesion, contraction and retraction (Ridley et al., 2003). During the protrusion step of cell migration, the cell generates two major types of actin-based structures: lamellipodia and filopodia. While filopodia are characterized by parallel, unbranched actin filaments (Svitkina et al., 2003), the lamellipodium is composed of a densely-branched network of actin filaments forming a sheet-like exploratory organelle (Abercrombie et al., 1970). One protein that defines the lamellipodia is the actin-related protein 2/3 (Arp2/3) complex which generates new branches from the sides of pre-existing filaments resulting in a highly branched actin network (Machesky et al., 1999; Mullins et al., 1997; Suraneni et al., 2012; Svitkina and Borisy, 1999). It is the addition of actin subunits (G-actin), spread across the entire expanse of the lamellipodium that leads to protrusion of this organelle. The Arp2/3 complex must be activated by proteins known as nucleation promoting factors (NPFs) in order to nucleate filaments (Machesky et al., 1999; Prehoda et al., 2000; Zalevsky et al., 2001). NPFs have been divided into two types: the WASP/N-WASP and the SCAR/WAVE family of proteins comprise type I NPFs, while Cortactin and the closely related hematopoetic-specific protein-1 (HS1) comprise type II NPFs (Goley and Welch, 2006). While type I NFPs generally bind and activate Arp2/3 via a shared VCA (verprolin homology, central, acidic) region, Cortactin and HS1 use an N-terminal acidic region (NtA) (Goley and Welch, 2006; Weaver et al., 2001).

Cortactin, unlike type I NPFs, can be found integrated within the lamellipodia. Data from fluorescence recovery after photobleaching (FRAP) analysis suggests that it recovers throughout the organelle after photobleaching rather than just at the leading edge (Lai et al., 2008). Cortactin can bind to both the sides of actin filaments and at Arp2/3-generated branch junctions where it is thought to stabilize them (Weaver et al., 2001). Interestingly, *in vitro* single-molecule experiments determined that Cortactin has a ∼300-fold increased affinity for branch junctions over the sides of actin filaments, suggesting the protein preferentially targets these sites (Helgeson and Nolen, 2013). Type I NPFs are more potent activators of the Arp2/3 complex than Cortactin, however the addition of Cortactin to GST-VCA beads increased bead motility, suggesting that Cortactin may synergize with type I NPFs during filament nucleation (Helgeson and Nolen, 2013; Siton et al., 2011; Weaver et al., 2002). Previously, it had been shown that Cortactin competes with the VCA domain for binding to the Arp3 subunit of the Arp2/3 complex, and more recently single-molecule experiments from Helgeson and Nolen demonstrate that Cortactin replaces the VCA domain of type I NPFs during nucleation (Helgeson and Nolen, 2013; Weaver et al., 2001). Thus, it appears that Cortactin both stimulates the formation of branches while simultaneously stabilizing them. This type of synergy may allow for continued dendritic nucleation while preventing the potential stalls caused by the tight membrane association of type 1 NPFs (Helgeson and Nolen, 2013). An examination of this synergy between type I and type II NPFs remains to be fully investigated *in vivo,* thus it is unclear how it fits into the paradigm of lamellipodial protrusion and cell migration.

Overexpression of Cortactin has been associated with increased metastasis and invasion in a number of cancers (Åkervall et al., 1995; Buday and Downward, 2007; Hirakawa et al., 2009; Kirkbride et al., 2011; Rothschild et al., 2006; Weaver, 2008; Xu et al., 2010). In support of this, overexpression of Cortactin in NIH 3T3 cells led to an increase in motility and invasiveness. Similarly, overexpression of Cortactin in breast cancer cells led to increased metastasis in nude mice (Patel et al., 1998). RNAi experiments in HT1080 cells suggest that Cortactin enhances lamellipodial persistence, and both the Arp2/3 and F-actin binding sites of Cortactin were required for this persistence (Bryce et al., 2005). Cortactin depletion also led to a decrease in the rate of adhesion formation, however, given the importance of lamellipodia to the formation of nascent adhesions, it may be difficult to uncouple these phenotypes (Bryce et al., 2005; Wu et al., 2012). Interestingly, studies from Lai and colleagues, which used cells-derived Cortactin-knockout mice, found few differences between the lamellipodia of Cortactin-null and wild-type fibroblasts. They observed a slight decrease in the assembly of actin in lamellipodia of Cortactin-null fibroblasts, as well as a decrease in the speeds of random cell migration and wound healing in scratch-wound assays. They also observed defects in PDGF-stimulated actin re-organization (Lai et al., 2009). These seemingly contradictory findings suggest that Cortactin's role in lamellipodial organization and actin dynamics still remains ill-defined.

Cortactin also localizes to other parts of the cell where dynamic actin assembly occurs including endosomes, podosomes, invadopodia and the dendritic spines of neurons (Ammer and Weed, 2008; Buday and Downward, 2007; MacGrath and Koleske, 2012; Ren et al., 2009). Coincident with Cortactin at some of these sites of dynamic actin are two Cortactin-binding proteins, Cortactin-binding protein 2 (CTTNBP2) and Cortactin-binding protein N-terminal-like (CTTNBP2NL or CortBP2NL).

Human CTTNBP2, coded for by the *CTTNBP2* gene, is found primarily in neurons. CTTNBP2 interacts with the C-terminal SH3 domain of Cortactin (Ohoka and Takai, 1998) and previous studies have demonstrated that CTTNBP2 co-localizes with both Cortactin and actin at lamellipodia. CTTNBP2 depletion in rat hippocampal neurons decreased the width and density of dendritic spines, suggesting that CTTNBP2 plays a role alongside with Cortactin in dendritic spine maintenance (Chen and Hsueh, 2012). Additionally, before dendritic spine formation, CTTNBP2 associates with microtubules through its central region and oligomerizes through its N-terminal region coiled-coil motif. CTTNBP2 oligomers bound to microtubules promotes microtubule bundle formation and tubulin acetylation (Shih et al., 2014).

Much less is known about CTTNBP2NL, and a clear cellular function for the protein has yet to be fully elucidated. CTTNBP2NL is found in epithelial, spleen and liver cells and unlike CTTNBP2, CTTNBP2NL does not associate at the cell cortex, but instead can be found on actin stress fibers where it can redistribute Cortactin to these structures (Chen et al., 2012). Interestingly, in rat hippocampal neurons, exogenous CTTNBP2NL is unable to rescue the effects of CTTNBP2 depletion on dendritic spine morphology (Chen et al., 2012), indicating that the two proteins are not functionally similar in the context of mammalian dendritic spine morphology. Given Cortactin's widespread expression, CTTNBP2NL may very well play important roles in other dynamic actin-based structures in non-neuronal cell types. *Drosophila* CG10915 is an uncharacterized protein-coding gene that shows amino acid sequence similarity to CTTNBP2 and CTTNBP2NL. CG10915 is expressed ubiquitously throughout the larval and adult fly, with higher expression levels in the central nervous system and ovaries (Gelbart and Emmert, 2013). Here, we investigate the role of CG10915 in *Drosophila* to determine its role in actin dynamics. We demonstrate that the *Drosophila* gene CG10915 [hereafter referred to as *Nausicaa* (*naus*)] alters lamellipodial and protrusive actin dynamics in migratory cells and neurons in a Cortactin-dependent manner.

## RESULTS

Bioinformatic queries indicated that the *Drosophila* CG10915 locus at cytological position 55B9 was a potential homolog of human Filamin-A interacting protein due to it sharing approximately 20% identity. Further refinement of these queries (Clustal Omega Multiple Sequence Alignment, Goujon et al., 2010; Sievers et al., 2011) indicated that CG10915 is similar to human CTTNBP2NL and CTTNBP2 sharing approximately 30 and 28% identity, respectively (Fig. S1). We have subsequently named CG10915, the putative *Drosophila* homolog of CTTNBP2 and CTTNBP2NL, *naus*, after the princess in Homer's *The Odyssey*, who helps to ensure Odysseus's safe passage home from Phaeacia. *Naus* has two splice variants (*cg10915*-*A* and *cg10915*-*B*), which vary in length in their 3′ and 5′-untranslated regions, however each encodes for an identical polypeptide 609 amino acids in length. The highest degree of conservation between Naus, CTTNBP2 and CTTNBP2NL occurs in the coiled-coil motif found within the N-terminal Cortactin-binding protein (CortBP2) domain. Furthermore, the three proteins also share a highly conserved proline-rich patch located near their C-termini that has been shown to facilitate the interaction between Cortactin and CTTNBP2 in COS cells (Fig. S1) (Chen et al., 2012).

### Naus localizes to lamellipodia of S2R+ cells in a Cortactin-dependent manner

**Fig. 1.**
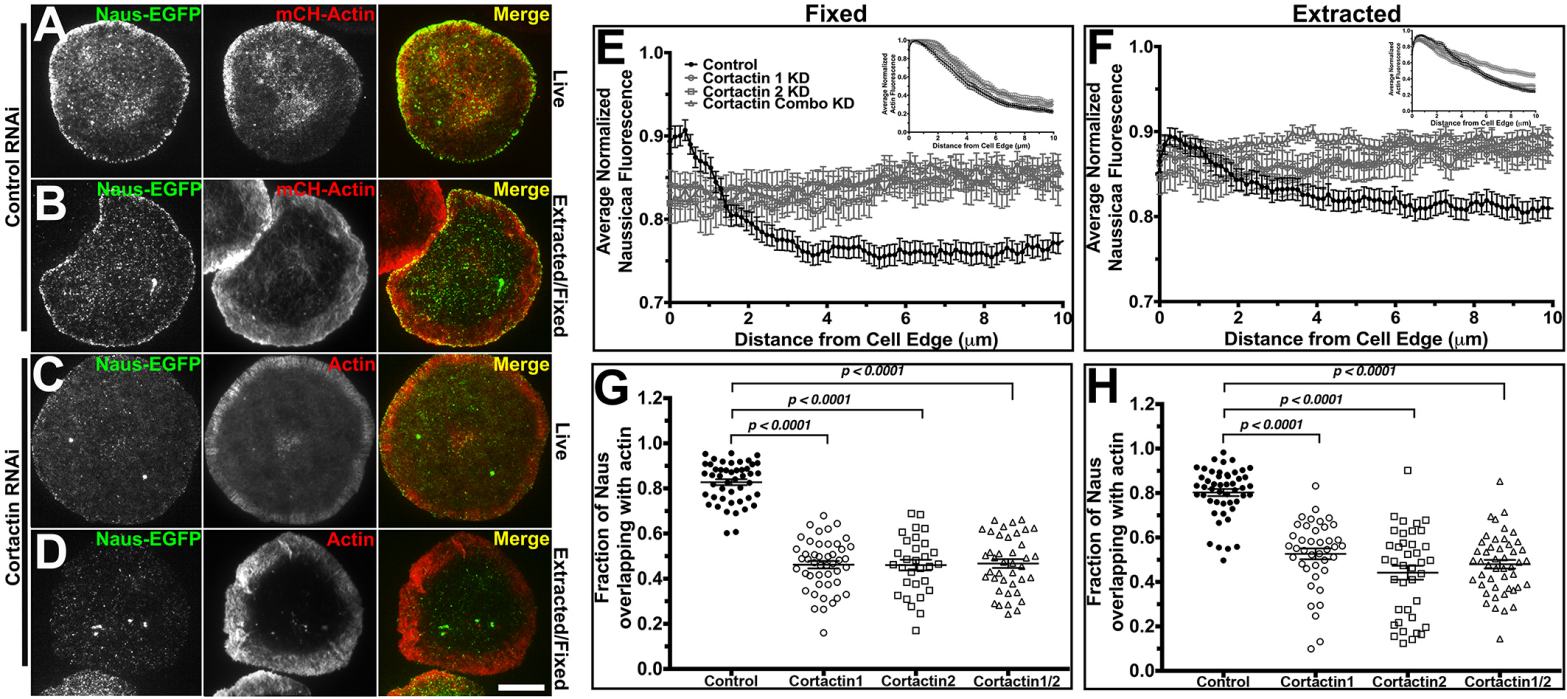
**Naus's** **lamellipodial localization is Cortactin-dependent.** (A,B) Control RNAi-treated S2R+ cells transfected with Naus-EGFP (left, green in merged image) and mCherry-Actin (middle, red in the merged image) imaged live (A) or extracted stained for F-actin (middle, red in the merged image) (B). (C,D) Cortactin RNAi-treated S2R+ cells transfected with Naus-EGFP (left, green in the merged images) and mCherry-Actin (middle, red in the merged image) imaged live (C) or extracted cells stained for (middle, red in the merged images) F-actin (D). Scale bar: 10 µm. (E) Line-scan analysis of the lamellipodial distribution of Naus-EGFP in fixed S2R+ cells treated with control RNAi (black circles) or Cortactin RNAi from two independent RNAi targets (open gray circles or squares) or the combination of the two targets (open gray triangles). Inset is the corresponding averaged normalized actin fluorescence. Error bars denote s.e.m. (F) Line-scan analysis of the lamellipodial distribution of Naus-EGFP in extracted cells following control RNAi (black circles) or treatment with two independent Cortactin RNAi targets (open gray circles or squares) or the combination of the two (open gray triangles). Inset is the corresponding averaged normalized actin fluorescence. Error bars denote s.e.m. (G) Mander's coefficient of the fraction of overlap between Naus-EGFP and F-actin stained by phalloidin. Cells were treated with control RNAi (black circles) or one of two Cortactin RNAi targets (open black circles or squares) or the combination of the two (open black triangles). Error bars show s.e.m., *n*=30–45 cells per condition; *P*<0.0001, Student's *t*-test). (H) Mander's coefficient quantifying the amount of overlap between Naus-EGFP and F-actin as visualized by phalloidin in extracted cells. Control RNAi is shown in black circles, Cortactin RNAi (from two independent targets) is shown in open black circles or squares and the combination of the two Cortactin RNAi targets is shown in open black triangles. Error bars show s.e.m., *n*=40–50 cells per condition; *P*<0.0001, Student's *t*-test.

We first assessed the localization of EGFP-tagged Naus by live-cell imaging of *Drosophila* S2R+ cells using total internal reflection fluorescence (TIRF) microscopy (Fig. 1A, Movie 1). Interestingly, we observed an enrichment of Naus in the circumferential lamellipodia of these cells which persisted following fixation (data not shown) and when we extracted the cells with detergent prior to fixation (Fig. 1B). This circumferential lamellipodial localization can also be observed by spinning disk confocal microscopy (Fig. S2A,B). We next investigated whether Naus localized to other actin-based structures. *Drosophila* S2R+ cells do not form prominent stress fibers, however, ML-DmD25c2 (D25) cells, which are derived from third instar imaginal wing discs, readily form these structures. We co-expressed EGFP-tagged Naus with mCherry-Alpha-actinin to mark stress fibers and actin bundles, and observed Naus weakly localizing to these structures as well (Fig. S3A). Furthermore, much like our results from S2R+ cells, we also observed lamellipodial enrichment of Naus in D25 cells (Fig. S3B). Interestingly, while CTTNBP2NL can also be found co-localizing to microtubules in COS cells (Chen et al., 2012), we failed to observe any colocalization between Naus and microtubules in either S2R+ or D25 cells under these conditions (data not shown). Collectively, these results suggest that Naus behaves similarly to both CTTNBP2 and CTTNBP2NL, localizing to both the lamellipodia and bundled actin structures.

Given the potential interaction between Naus and *Drosophila* Cortactin (CG3637), we next tested whether this lamellipodial enrichment in S2R+ cells is Cortactin-dependent. While it has been demonstrated that mammalian CTTNBP2 and CTTNBP2NL interacts with Cortactin, the role Cortactin plays in this interaction is unclear. Using two independent dsRNA sequences, we depleted Cortactin (Fig. S4A) and expressed EGFP-tagged Naus and observed a distinct loss on Naus's lamellipodial localization (Fig. 1C, Movie 1). This loss in enrichment was even more evident in cells that were detergent extracted prior to fixation (Fig. 1D). Line-scan analysis where we compared control RNAi-treated cells to cells treated with either Cortactin dsRNAs or in combination, further corroborated this change in localization (Fig. 1E). To quantify this change, we used Mander's coefficient and measured the fraction of Naus overlapping with F-actin (stained by fluorescently labeled phalloidin) following Cortactin depletion and found a statistically significant decrease in the amount of Naus overlapping with actin, further supporting that Naus's association with actin cytoskeleton is Cortactin-dependent (Bolte and Cordelières, 2006) (Fig. 1G,H). This differs from CTTNBP2 where upon Cortactin re-distribution, CTTNBP2 does not re-localize in neurons suggesting a Cortactin-independent mechanism of localization for this potential Naus homolog (Chen and Hsueh, 2012). Given that Cortactin interacts with Arp2/3 complex at the lamellipodia (Uruno et al., 2001), we depleted the p20 subunit of Arp2/3 complex by RNAi and observed a similar loss of localization to the periphery of the cells (Fig. S5). Collectively, these results suggest that Naus is enriched in the lamellipodia and that this enrichment to actin structures is Cortactin-dependent.

**Fig. 2.**
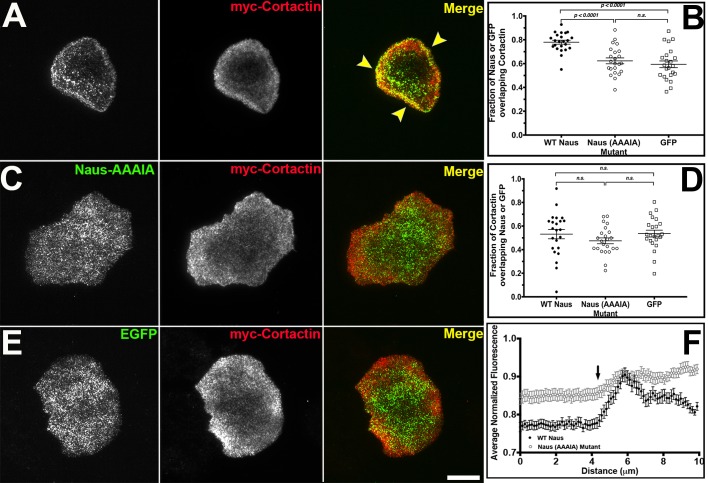
**Naus** **colocalizes with Cortactin through a conserved proline-rich motif.** (A,C,E) Fixed S2R+ cells co-transfected with myc-Cortactin (middle, red in merged image) and (A) Naus-EGFP, (C) Naus(AAAIA)-EGFP mutant and (E) EGFP control (left, green in merged images). Yellow arrowheads in A indicate regions of colocalization between Naus-EGFP and myc-Cortactin. Scale bar: 10 µm. (B) Mander's coefficient analysis for the fraction of Naus-EGFP or EGFP control overlapping with Myc-Cortactin (*P*<0.0001, one-way ANOVA with Tukey's multiple comparison post-hoc analysis; error bars show s.e.m., *n*=23 cells per condition). (D) Mander's coefficient analysis for the fraction Cortactin overlapping with Naus-EGFP or EGFP control. None of these differences were statistically significant by one-way ANOVA (n.s.). (F) Line-scan analysis of the lamellipodial distribution of WT Naus-EGFP (black circles) or Naus-EGFP AAAIA (open gray circles) in fixed S2R+ cells (error bars denote s.e.m., *n*=23 cells per condition). Arrow denotes the position of the cell edge.

Given that Naus's lamellipodial localization is Cortactin-dependent, we next wanted to characterize the relationship between the two proteins. We co-expressed Naus-EGFP with myc-tagged Cortactin in S2R+ cells and again used Mander's coefficient to determine the degree of overlap between these proteins (Fig. 2A,B,D). The Mander's coefficient revealed that just over 50% of myc-Cortactin overlapped with Naus-EGFP while nearly 80% of Naus overlapped with Cortactin. This asymmetry in co-localization, which was statistically significant (Student's *t*-test, *P*<0.0001), suggests that while not all of the Cortactin in the cell is associated with Naus, the majority of the Naus in the cell can be found overlapping with Cortactin. This supports the hypothesis that Naus relies on Cortactin for proper localization. Naus, like CTTNBP2 and CTTNBP2NL, has a proline-rich patch (PPPIP) that was previously shown to be required for Cortactin binding (Fig. S1) (Chen et al., 2012). To further elucidate the relationship between Naus and *Drosophila* Cortactin we mutated all of these proline residues (amino acid positions 563–567) to alanine and expressed an EGFP-tagged version (Naus-AAAIA) in S2R+ cells (Fig. 2C). Our initial observations indicated that rather than a specific localization to actin-based structures, Naus-AAAIA appeared to be distributed non-specifically throughout the cell which a pattern similar to what we observe when we expressed untagged-EGFP in these cells (Fig. 2C,E, Fig. S2C,D). Line-scan analysis corroborates this observation revealing distinct loss in lamellipodial-enriched Naus when these residues are mutated (Fig. 2F). This loss is similar to the loss of lamellipodial enrichment we observed following Cortactin RNAi (Fig. 1C). When we quantified the amount of colocalization by Mander's coefficient, we observed a statistically significant decrease in the amount of overlap between Naus-AAAIA and Cortactin further supporting the observation that this proline patch is facilitating the interaction between Naus and Cortactin (Fig. 2B,D). Similar to what we observed in S2R+ cells, EGFP-tagged Naus-AAAIA failed to localize specifically to actin structures in D25 cells (Fig. S3C). These results suggest that Naus, like its mammalian counterparts, interacts with Cortactin through this conserved proline patch, but uniquely, requires Cortactin for proper localization. Interestingly, while we failed to observe microtubule localization in cells expressing wild-type EGFP-Naus, on occasion we did observe EGFP-tagged Naus-AAAIA co-localizing with microtubules in both S2R+ and D25 cells (Fig. S3D). It is likely that under conditions where its affinity for Cortactin is reduced, Naus may bind microtubules. While more detailed analysis of this microtubule localization is needed, we feel that this is beyond the scope of this current study.

**Fig. 3.**
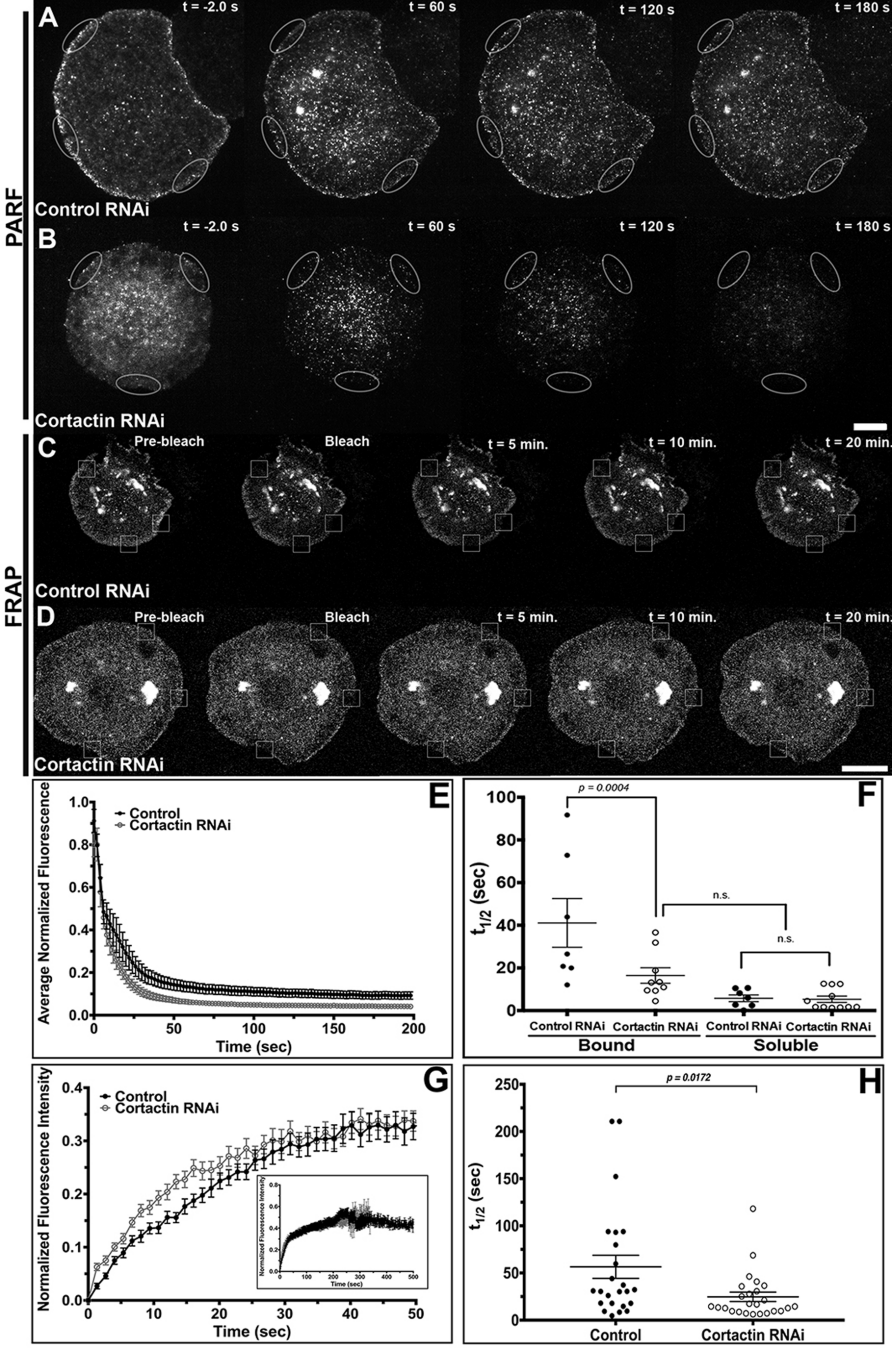
**Depletion of Cortactin alters Naus** **dynamics in the lamellipodia of S2R+ cells.** (A,B) Time-lapse images of PARF in S2R+ cells transfected with Naus-EGFP following control RNAi (A) or Cortactin RNAi (B) treatments. Gray ovals denote the regions where the PARF measurements were taken. For each cell, three separate regions were measured and averaged. (C,D) Time-lapse images of fluorescence recovery after photobleaching (FRAP) of S2R+ cells transfected with Naus-EGFP following (C) control RNAi or (D) Cortactin RNAi treatments. Small white boxes denote regions bleached. Scale bars: 10 µm. (E) Average normalized fluorescence decay from PARF experiments. Error bars denote s.e.m. (F) The average half-life of Naus-EGFP as determined by PARF. The data was fit to a two-phase (bound and soluble) exponential decay model, control (black circles) or Cortactin RNAi (open black circles), *P*=0.0004, one-way ANOVA with Tukey's multiple comparison post-hoc analysis, control: *n*=7 cells, Cortactin RNAi: *n*=11 cells, error bars show s.e.m. n.s., not significant (one-way ANOVA). The average R values for the two-phase (bound and soluble) exponential model were 0.962 and 0.942 for control and Cortactin RNAi conditions, respectively. (G) Average normalized fluorescence recovery from FRAP experiments, in black circles, control RNAi and open black circles, Cortactin RNAi. Error bars show s.e.m. (H) Average half-life of Naus-EGFP fluorescence recovery for control (black circles) or Cortactin RNAi (open gray circles)-treated cells from the FRAP experiments (*P*=0.0178, two-tailed Student's *t*-test, control: *n*=24 cells, Cortactin RNAi: *n*=25 cells, error bars show s.e.m.).

Given this dependence on Cortactin for its lamellipodial localization, we next sought to determine if Naus's dynamics are altered in the absence of Cortactin. We first used permeabilization activated reduction in fluorescence (PARF) to measure the loss of Naus-EGFP fluorescence following control or Cortactin RNAi treatments (Fig. 3). PARF uses a low concentration of digitonin to gently permeabilize cells leading to a large-scale dilution of the unbound pool of protein and a disruption of the initial equilibrium of the bound protein. The subsequent decrease in fluorescence can be fit to a two-phase exponential decay model which can be used to calculate a half-life (t_1/2_) for fluorescence loss for both the bound and soluble fractions (Singh et al., 2016). Cortactin depletion led to a rapid loss in Naus-EGFP fluorescence following permeabilization for both fractions with an average half-life of 16.44±3.62 s for the bound population and 5.33±1.48 s for the soluble fraction, while control RNAi-treated cells had an average half-life of fluorescence decay of 41.10±11.43 s for the bound fraction and 5.78±1.58 s for the soluble fraction. While the kinetics of the soluble fraction of EGFP-Naus was indistinguishable between RNAi conditions, the half-life of fluorescence decay for the control bound fraction was more than double that of Cortactin-depleted cells (Fig. 3A,B,E,F, Movies 2 and 3). Moreover, the half-life of fluorescence decay for the bound population EGFP-Naus in cells depleted of Cortactin was indistinguishable from that of the soluble unbound fraction in control RNAi-treated cells. These results suggest that depletion of Cortactin leaves a larger portion of the Naus pool free to quickly diffuse out of the cell rather than maintaining association with Cortactin and the actin cytoskeleton. To corroborate our PARF results, we also performed FRAP in cells treated with control or Cortactin RNAi. Again, we found that depletion of Cortactin led to a decrease in the half-life of recovery, from 56.5±12.2 s in control cells to 24.7±4.9 s in Cortactin-depleted cells (Fig. 3C,D,G,H, Movies 4 and 5). Similar to our PARF results, our FRAP experiments suggest that in the presence of Cortactin, Naus is more stably associated with the cytoskeleton leading to a slower half-life of recovery as compared to Cortactin-depleted cells. Collectively, these results indicate that Cortactin may function as an anchor, helping Naus maintain lamellipodial localization.

**Fig. 4.**
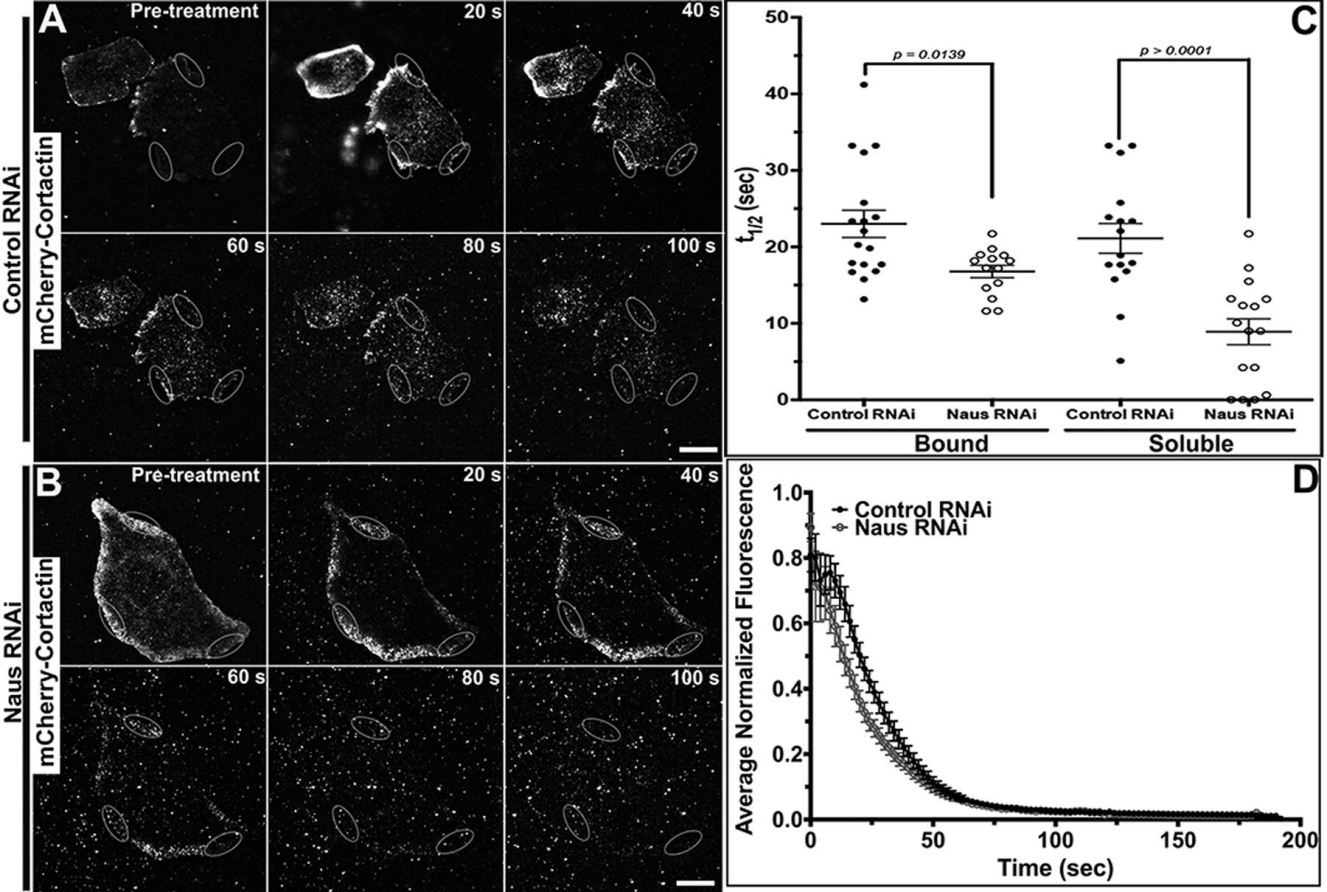
**Depletion of Naus** **alters Cortactin dynamics in S2R+ cells.** (A,B) Time-lapse images of PARF of S2R+ cells transfected with mCherry-Cortactin following control RNAi (A) or Naus RNAi (B) treatments. Gray ovals denote the regions where the PARF measurements were taken. For each cell, three separate regions were measured and averaged. Scale bars: 10 µm. (C) Average half-life of mCherry-Cortactin fluorescence decay obtained from a two-phase (soluble and bound) exponential model for control (black circles) or Naus RNAi (open black circles) treatments. The average R values for the two-phase model were 0.987 and 0.992 for control and Naus RNAi conditions, respectively (*P=*0.0139, Mann–Whitney test, control: *n*=10 cells, Naus RNAi: *n*=11 cells, error bars show s.e.m.). Half-life of fluorescence decay for the soluble fraction of mCherry-Cortactin (*P*>0.0001, Mann–Whitney test, control: *n*=10 cells, Naus RNAi: *n*=11 cells, error bars show s.e.m.). (D) Average normalized mCherry-Cortactin fluorescence decay from PARF experiments (control RNAi, black circles; Naus RNAi, open gray circles).

While our results indicate that Cortactin affects Naus's dynamics, we wanted to determine if the inverse is also true. Using PARF we measured the loss of Cortactin fluorescence following RNAi depletion of Naus (Fig. 4A,B, Fig. S4B). Again, we fit the PARF data to a two-phase exponential decay model separating the kinetics of bound and soluble fractions of mCherry-Cortactin. This analysis revealed that depletion of Naus led to a statistically significant (*P*=0.0139, Student's *t*-test, *n*=14–18 cells) decrease in the half-life of the bound fraction of mCherry-Cortactin as compared to control RNAi-treated samples (Fig. 4C,D, Movies 6 and 7). The average half-life of fluorescence decay for bound mCherry-Cortactin following Naus depletion was 16.72±0.82 s, while the half-life of fluorescent decay in control-treated cells was 23.01±1.8 s (Fig. 4C). Interestingly, Naus depletion also appears to decrease the half-life of fluorescence decay for the soluble fraction of mCherry-Cortactin as well. We observed a statistically significant decrease in the half-life of fluorescence decay for the soluble pool of mCherry-Cortactin from 21.11±1.9 s for control RNAi-treated samples to 8.905±1.7 s for Naus RNAi-treated samples (Fig. 4C). This decrease in half-life may reflect an overall increase in the amount of unbound mCherry-Cortactin as a result of Naus depletion. We observed a similar increase in Cortactin's mobility by PARF when we used RNAi targeting the 5′UTR of Naus (data not shown). These findings are also in line with the increase in Cortactin's mobility in the dendritic spines of rat primary hippocampal neurons depleted of CTTNBP2 following FRAP analysis (Chen et al., 2012).

### Depletion of Naus alters lamellipodial actin dynamics

**Fig. 5.**
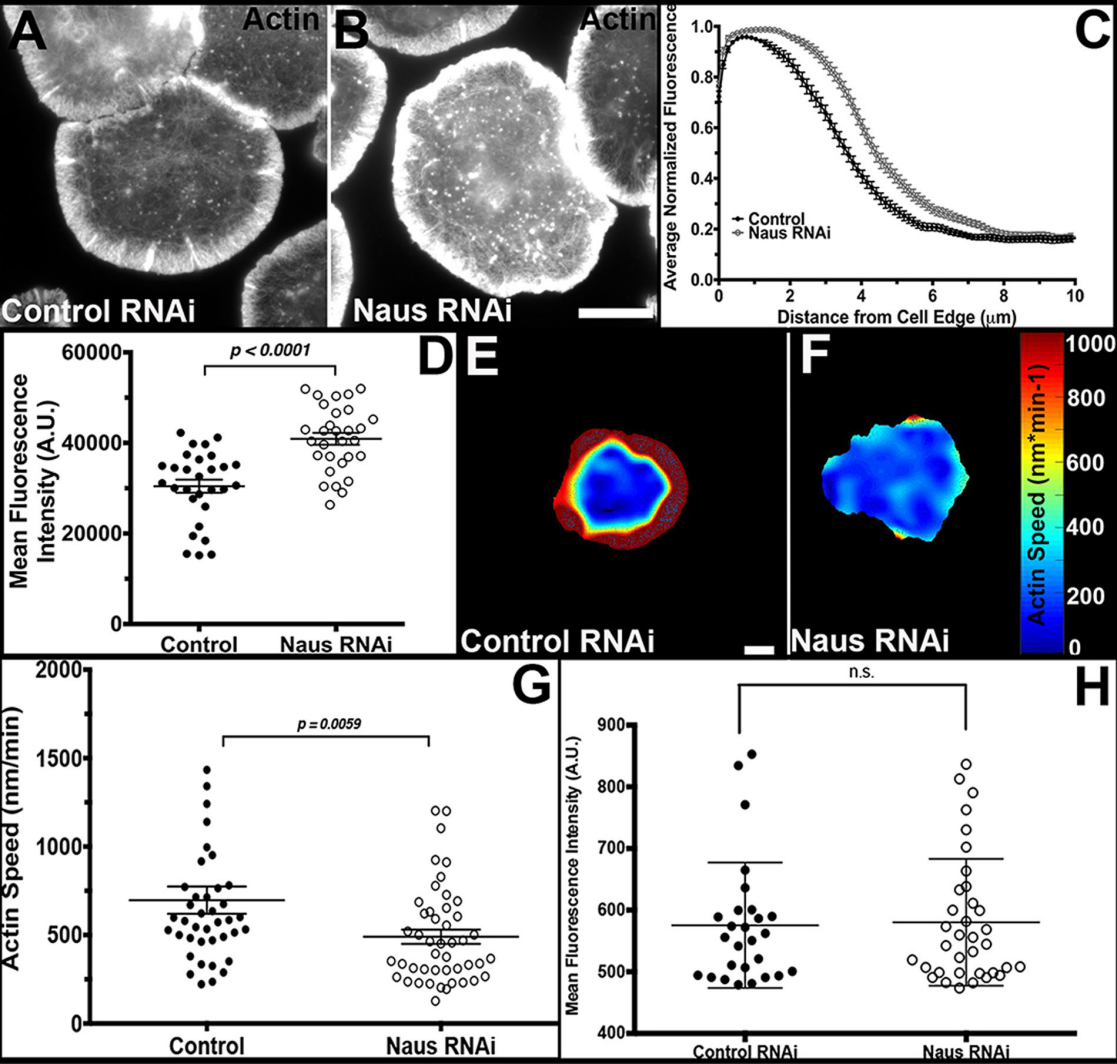
**Naus** **regulates lamellipodial actin density and actin retrograde flow in S2R+ cells.** (A,B) Fixed S2R+ cells treated with control (A) or Naus RNAi (B) stained for F-actin with phalloidin. Gray levels have been set equal for comparison. Scale bar: 10 µm. (C) Line-scan analysis of F-actin fluorescence in the lamellipodia from cells as shown in A and B. Fluorescence was normalized for each cell and averaged for each condition (black circles, control RNAi and open gray, circles Naus RNAi). (D) Mean fluorescence intensity of lamellipodial actin of cells treated with control RNAi (black circles) or Naus RNAi (open black circles) from cells as shown in A and B (*P*<0.0001, Student's *t*-test, *n*=30 cells per condition). (E,F) Representative heat maps of actin speeds in (E) control or (F) Naus RNAi-treated cells from QFSM analysis. Cool colors indicate slower rates of retrograde flow and warm colors represent faster speeds of actin retrograde flow. Scale bar: 10 µm. (G) Quantification of lamellipodial actin speeds from QFSM analysis (*P=*0.0059, Mann–Whitney test, control RNAi: *n*=40 cells, Naus RNAi: *n*=46 cells). (H) Quantification of the mean fluorescence intensity of EGFP-Actin in the cells analyzed by QFSM (black circles, control RNAi and open black circles, Naus RNAi), n.s., not significant (Mann–Whitney test).

Given the putative role Naus plays in regulating Cortactin dynamics, we sought to determine if Naus plays a role in regulating lamellipodial actin dynamics. We first depleted Naus in S2R+ cells and examined the circumferential lamellipodia of these cells by quantitative fluorescence microscopy (Fig. 5A–D). Using phalloidin to measure F-actin we performed line-scan analysis and quantified the mean actin density of the lamellipodia (Fig. 5C,D). Our analysis revealed an increase in actin fluorescence in the lamellipodia following Naus depletion which was statistically significant (*P*<0.001, Student's *t*-test, *n*=30 cells per condition) when compared to control RNAi-treated cells prepared in parallel (Fig. 5D).

As this increase in filamentous actin likely implies a change in dynamics, we next asked if depletion of Naus leads to changes in the rates of actin retrograde flow. To measure actin retrograde flow we turned to quantitative fluorescence speckle microscopy (QFSM) (Danuser and Waterman-Storer, 2006). Following treatment with Naus or control RNAi, we transfected S2R+ cells with EGFP-tagged actin driven by a copper-inducible promoter which allowed us to closely regulate the level of expression (Iwasa and Mullins, 2007). We imaged the cells by TIRF microscopy and analyzed the resulting movies using a Matlab-based program, QFSM, developed by the Danuser lab (Fig. 5E,F, Movie 8) (Mendoza et al., 2012). Interestingly, Naus depletion led to a statistically significant 1.4-fold (*P*=0.0059, Student's *t*-test, *n*=40 cells and 46 cell for control and Naus RNAi, respectively) decrease in actin retrograde flow speeds in the lamellipodia as compared to control RNAi-treated cells (Fig. 5G, Fig. S6). We measured the mean fluorescence intensity of EGFP-actin in these cells to determine if actin expression levels were dictating the speed of retrograde flow and found no statistically significant difference between the two RNAi conditions (Fig. 5H). This slowing of actin dynamics in combination with an increase in F-actin in the lamellipodia indicates that Naus helps to regulate actin branch dynamics, likely through its interaction with Cortactin.

### Depletion of Naus leads to an increase in the number of long unbranched actin filaments in the lamellipodia

**Fig. 6.**
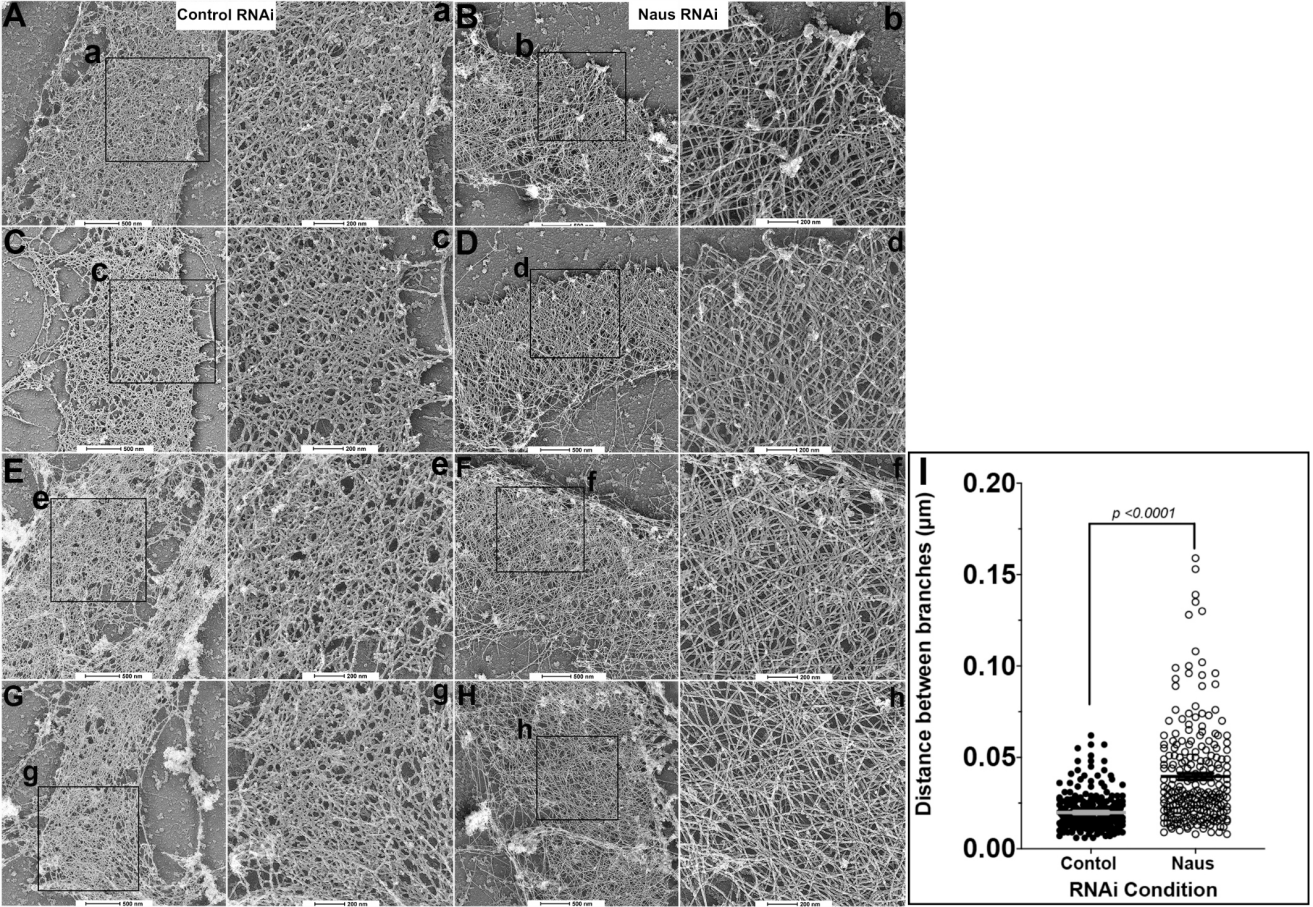
**Depletion of Naus leads to an increase in the number of long unbranched actin filaments on the lamellipodia.** Platinum replicas of the lamellipodia of S2R+ cells treated with control (A,C,E,G) or Naus (B,D,F,H) RNAi. The black box denotes the region shown at higher magnification (control RNAi: a,c,e,g and Naus RNAi: b,d,f,h). Scale bars: (higher magnification images) 200 nm, (lower magnification images) 500 nm. (I) Quantification of branch length from the images in a–h (*P*<0.0001, Students *t*-test, *n*>40 measurements were made per image, across five separate images for each condition, bars represent mean and s.e.m.).

The decrease in the rate of actin polymerization coupled with the increase in filamentous actin we observed in the lamellipodia of Naus-depleted S2R+ cells suggests that Naus may play a role in regulating the fundamental architecture the of actin cytoskeleton. To test this, we turned to platinum replica electron microscopy (Svitkina, 2016). We generated platinum replicas of control and Naus-depleted S2R+ cells and imaged the actin cytoskeleton of the lamellipodia using electron microscopy (Fig. 6). Interestingly, while the lamellipodia of control-treated cells remained highly branched, typical of an Arp2/3 nucleated dendritic network (Fig. 6A,C,E,G), the lamellipodia of Naus-depleted cells was composed of extremely long, unbranched filaments with very few branch junctions (Fig. 6B,D,F,H). We quantified the distance between actin branch points or filament intersections and found a statistically significant increase in this length following Naus depletion (Fig. 6I). Given that Cortactin is both an NPF and can stabilize the Arp2/3 complex at branch junctions, these long unbranched filaments we observed are likely the result of a reduction in Cortactin's activity. Without the stabilization provided by Naus, Cortactin fails to remain associated with Arp2/3 branches ultimately leading to reductions in actin polymerization and branch formation. Furthermore, these data assert a novel role for Naus in the regulation of the lamellipodial machinery and likely has broader implications for the way this actin-based protrusive organelle functions during cell migration.

### Depletion of Naus and Cortactin leads to a decrease in the lamellipodial localization of the Arp2/3 complex

**Fig. 7.**
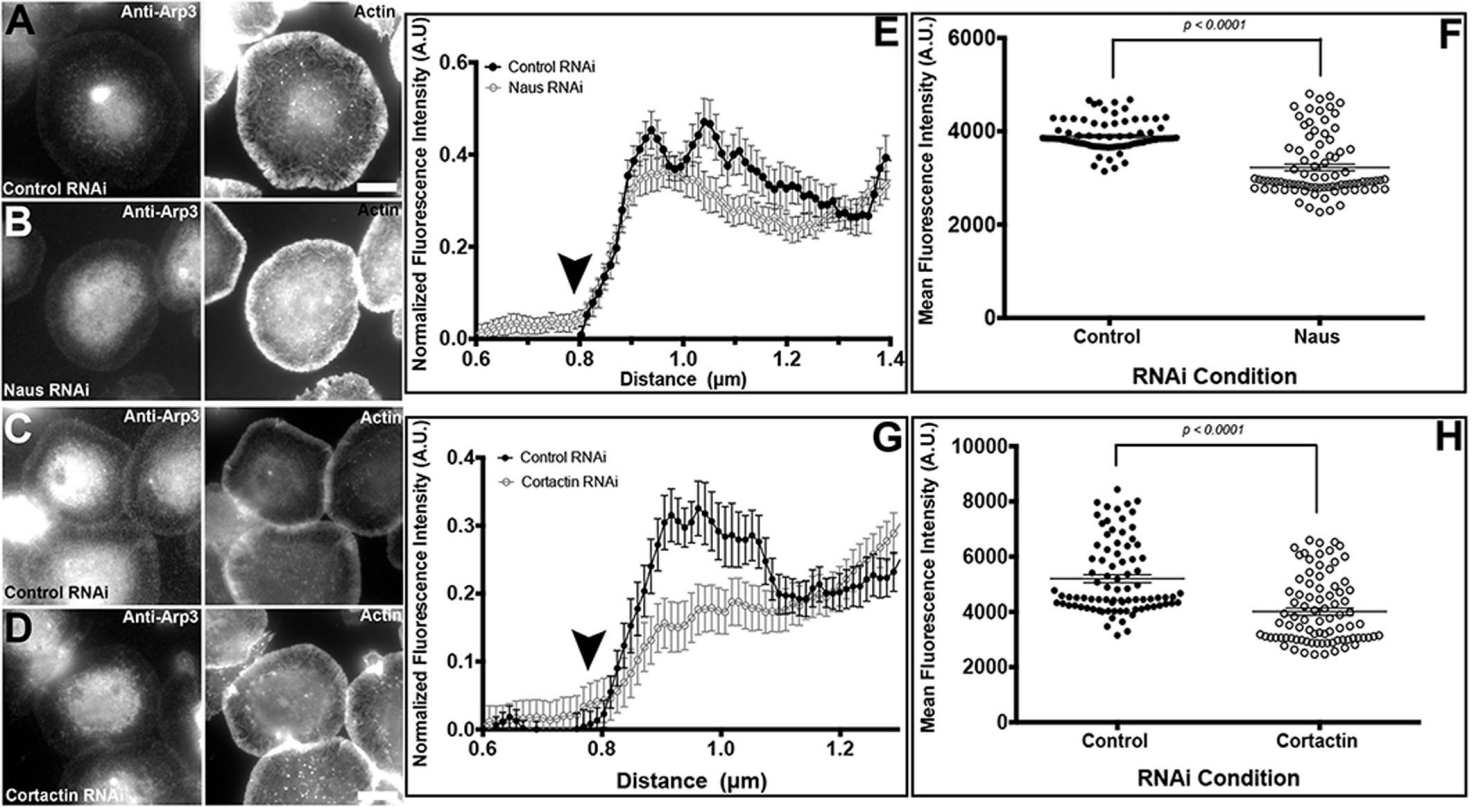
**Depletion of Naus and Cortactin leads to a decrease in Arp2/3 throughout the lamellipodia of S2R+ cells.** (A–D) S2R+ cells treated with (A,C) control, (B) Naus or (D) Cortactin RNAi were fixed and stained for the Arp3 subunit of the Arp2/3 complex (Anti-Arp3) and F-actin (Actin) with phalloidin. Gray levels have been set equal for comparison. Scale bars: 10 µm. (E,G) Line-scan analysis of Arp3 fluorescence in the lamellipodia from cells as shown in A–D. Fluorescence was normalized for each cell and averaged for each condition (black circles, control RNAi; open gray circles, Naus RNAi or Cortactin RNAi) error bars show s.e.m. The black arrowheads indicate the cell edge. (F,H) Mean fluorescence intensity of Arp3 of cells treated with control RNAi (black circles) or Naus RNAi or Cortactin (open black circles) from cells as shown in A–D. Error bars are s.e.m. (*P*<0.0001, Mann–Whitney test, *n*=8–10, three independent measurements per cells per condition).

Our platinum replica results prompted us to examine the localization of the Arp2/3 complex in Naus-depleted S2R+ cells. Following a 7-day treatment of Naus, Cortactin and control RNAi, cells were fixed and stained using an antibody generated against the Arp3 subunit of the Arp2/3 complex (Fig. 7). We next performed a series of quantitative line scans to measure the fluorescence of Arp3 throughout the circumferential lamellipodia of these cells. Intriguingly, we observed a decrease in Arp3 staining throughout the lamellipodia of Naus and Cortactin-depleted cells as compared to controls (Fig. 7A–G) and when we measured the average fluorescence intensity across the lamellipodia we found a statistically significant decrease in Arp3 staining (*P*<0.0001, Mann–Whitney test, *n*=8–10 cells, three independent measurements per cell). These results taken together with our ultrastructural data further suggest that depletion of Naus leads to a reduction in Arp2/3 branching in the lamellipodia likely through the precocious dissociation of Cortactin from branch junctions. While our experimental set-up prevents us from directly comparing Naus and Cortactin-depleted cells, there does appear to be a greater reduction in Arp3 staining following Cortactin depletion as compared to control cells than Naus-depleted cells. This observation further corroborates our hypothesis in that Cortactin stabilizes Arp2/3 branches and Naus functions to delay dissociation or anchor Cortactin.

### Depletion of Naus leads to a decrease in cell migration and directionality

**Fig. 8.**
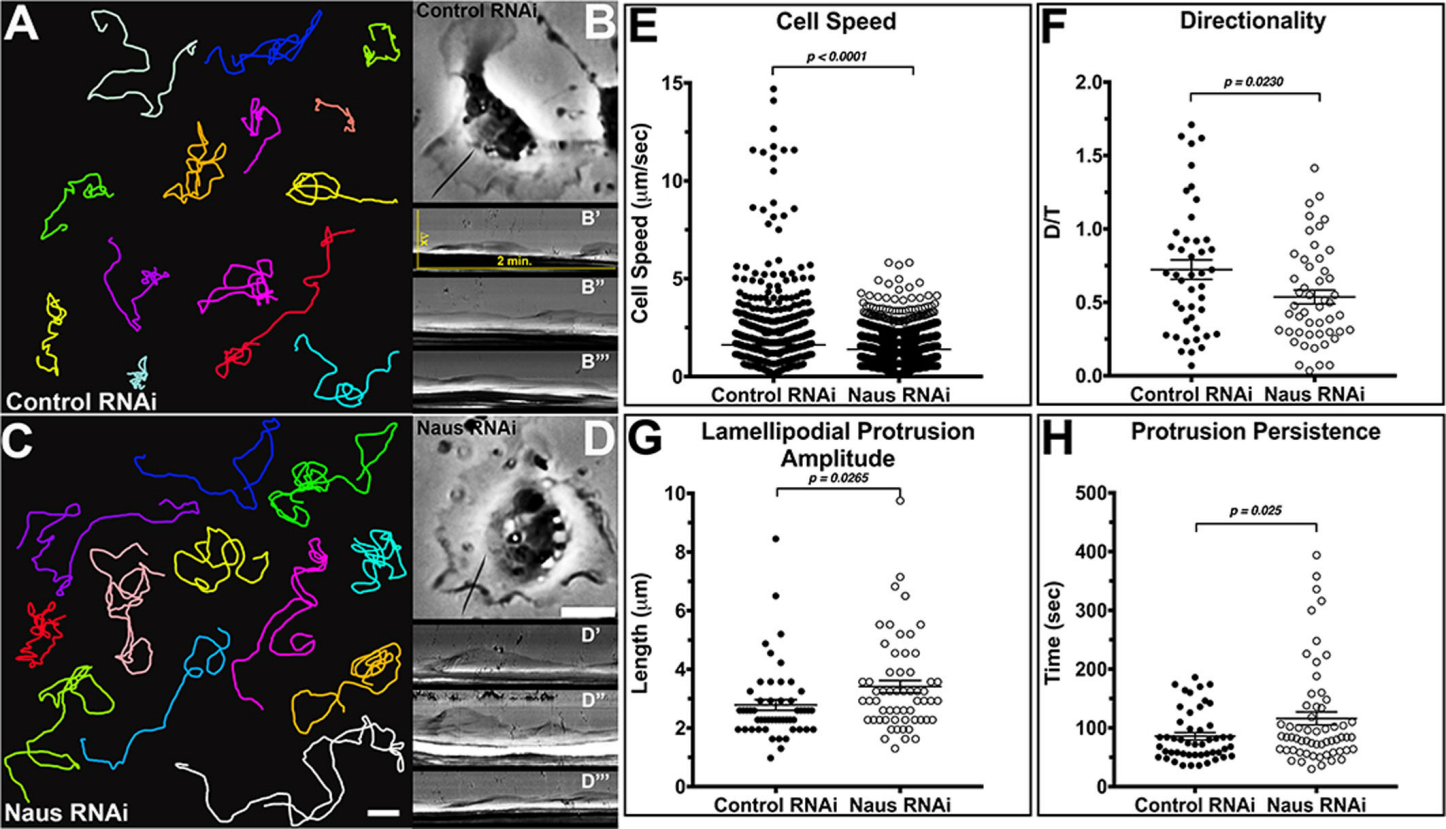
**Naus** **regulates directionality and lamellipodial persistence in migrating D25c2 cells.** (A–D) Representative migration tracks for *Drosophila* D25 cells treated with (A,B) control or (C,D) Naus RNAi. (B′–B‴) Representative kymographs from control RNAi-treated D25 cells; the black line represents a typical region of interest used to generate kymographs. (D′–D‴) Representative kymographs for Naus RNAi-treated D25 cells, the black line represents a typical ROI used to generate kymographs. Scale bars: 10 µm. (E) Average instantaneous cell speeds for control (black circles) or Naus RNAi (open black circles)-treated D25 cells. (*P*<0.0001, two-tailed Student's *t*-test; control: *n*=43 cells, 1438 measurements; Naus RNAi: *n*=50 cells, 1779 measurements). (F) Quantification of directionality, between control (black circles) and Naus (open black circles) measured as the ratio of D/T where D is distance between starting and end point and T is the total distance traveled. (*P*=0.0230, two-tailed Student's *t*-test, control: *n*=44 cells, Naus RNAi: *n*=49 cells). (G) Quantification of lamellipodial protrusions following control (black circles) or Naus RNAi (open black circles)-treated D25 cells (*P*=0.0265, two-tailed Student's *t*-test, control: *n*=51 cells, Naus RNAi: *n*=58 cells). (H) Quantification protrusion persistence in Naus RNAi (open black circle) or control (black circles)-treated D25 cells (*P*=0.025, two-tailed Student's *t*-test, control RNAi: *n*=49 cells, Naus RNAi: *n*=59 cells). All error bars show s.e.m.

Actin polymerization is the main engine behind lamellipodial protrusion and ultimately, cell migration. Given the alterations to both actin dynamics and architecture we observed following Naus depletion, we next sought to determine whether Naus plays a role in cell migration. To do this, we performed a random cell migration assay where we treated D25 cells with control or Naus RNAi for 7 days, plated them on a mixture of extracellular matrix (ECM) and imaged them by phase-contrast microscopy for 6 h (Fig. 8A–D, Movie 9). Cells were then manually tracked yielding migration speeds. In comparing the instantaneous velocity of approximately 50 cells per condition we found that depletion of Naus led to a modest but statistically significant decrease in the speed of cell migration. Control RNAi cells migrated at an average rate of approximately 1.6±0.09 μm min^−1^, while Naus-depleted cells migrated at an average rate of 1.4±0.05 μm min^−1^(*P*<0.0001, Student's *t*-test) (Fig. 8E). Even during random migration, cells will still maintain a degree of directionality. When we measured the directionality of Naus-depleted cells we found they were statistically significantly less directional than control RNAi-treated cells. Where a value of 1.0 is completely directional, control RNAi-treated cells showed a value of 0.7±0.07 a.u. (arbitrary units) while Naus-depleted cells were 0.5±0.04 a.u. (*P*=0.0230, Student's *t*-test) (Fig. 8F). Directional persistence is a function of actin branch density and the density of actin branches positively correlates with the directionality of randomly migrating cells (Harms et al., 2005). Thus, the decrease in directionality we observed in Naus-depleted D25 cells is consistent with the ultrastructural data gathered from S2R+ cells.

While these results suggest that Naus plays a role in maintaining both the speed and the directionality of migrating cells, we wanted the further explore how Naus regulates the lamellipodial dynamics that govern cell migration. Using kymographs taken from phase-contrast microscopy movies we measured lamellipodial persistence, the speed of protrusion and retraction, the frequency of protrusions and the amplitude of protrusions (Fig. 8B,D) (Bear et al., 2002; Hinz et al., 1999). Despite the slower rates of cell migration, Naus-depleted cells had longer lamellipodial protrusions that persisted for greater periods of time as compared to control RNAi-treated cells. The average maximum length of lamellipodial protrusions (the amplitude of protrusions) for Naus-depleted D25 cells was 3.4±0.21 μm, which is statistically significantly greater than control cells at 2.8±0.18 μm (*P*=0.0265, Student's *t*-test) (Fig. 8G). These data corroborate our ultrastructural data in S2R+ cells and suggest the lamellipodia of Naus-depleted D25 cells may also contain longer unbranched filaments. Interestingly, unlike the less persistent lamellipodia of cells where Enabled (Ena)/VASP proteins were membrane targeted, we observed an increase in persistence following Naus depletion, from 85.8±6.0 s in control RNAi-treated cells to 116±11.0 s in Naus RNAi-treated cells (*P*=0.025, Student's *t*-test) (Fig. 8H). This suggests that despite having long unbranched filaments, the lamellipodia of Naus-depleted cells are still able to protrude without an increase in buckling. Depletion of Naus had a specific effect on lamellipodial dynamics, however other parameters such as the frequency of protrusions, the speed of protrusions and retractions, and the total distance the cells migrated over a 2-h period remain unchanged (Fig. S7). Collectively, we found that Naus depletion in D25 cells led to an increase in lamellipodial persistence while simultaneously decreasing cell migration speeds and directionality.

### Depletion of Naus decreases the number of branches in *Drosophila* larval neurons

This fine-tuning of actin dynamics is not only critical to the function of lamellipodia, but plays a major role in the morphology and function of other dynamic actin structures. One actin based-structure that is particularly sensitive to changes in actin dynamics are dendritic spines (Fischer et al., 1998; Hotulainen and Hoogenraad, 2010; Matus, 2000). Accordingly, we sought to determine whether Naus also plays a role in morphology of *Drosophila* neurons. While it remains controversial whether *Drosophila* neurons form dendritic spines *in vitro* we focused on the overall neuronal morphology of third instar larvae neurons in culture. Using the UAS-Gal4 system, we depleted Naus specifically in neurons using the pan-neuronal Gal4 driver Elav. The brains of third instar larvae were removed and enzymatically dissociated. The resulting neuroblasts were allowed to differentiate in culture for 24 h (Lu et al., 2013) (Fig. S8A–C). Following fixation and staining with the neuronal marker Futsch, the morphology of the neurons was assessed using Sholl analysis (Ferreira et al., 2014; Sholl, 1953). Sholl analysis quantifies the neuronal morphology by counting the number of intersections of concentric circles from the center of the cell body. Interestingly, we observed a distinct difference in the Sholl profiles of Naus-depleted neurons as compared to control neurons prepared in parallel, suggesting a difference in neuronal arborization (Fig. S8D). Similarly, the maximum Sholl radius in which neuronal intersections were still detected was significantly lower in Naus-depleted neurons (Fig. S8E). Consistent with this result, when neuron 2D skeletons were analyzed with ImageJ Simple Neurite Tracer (Longair et al., 2011), we observed that Naus-depleted neurons showed decrease in the number of branches as compared to controls (Fig. S8F,G). Taken together, these results suggest that Naus plays a role in neuronal branch arborization.

## DISCUSSION

The formation of actin protrusive structures such as the lamellipodia of migrating cells and the dendritic spines of neurons relies on Arp2/3 generated actin branches. Changes to the density and stability of actin branches can affect the overall morphology of these structures and ultimately, their function. Here we characterize Naus, a putative *Drosophila* homolog of two mammalian Cortactin binding proteins, CTTNBP2 and CTTNBP2NL. Using cultured and primary *Drosophila* cells we demonstrate that Naus, through its interaction with Cortactin, regulates actin-branch dynamics, lamellipodial protrusion and the morphology of neurons.

### Naus is likely the fly homolog of both CTTNBP2 and CTTNBP2NL

We described a previously uncharacterized protein encoding gene *cg10915*, which we have subsequently named *naus*. While bioinformatic queries indicate the *naus* is more closely related to mammalian CTTNBP2NL than CTTNBP2, based on its localization and putative role in regulating the morphology of neurons, we argue that Naus covers the function for both proteins in flies. We do not have to look much further for another example of this refinement of the *Drosophila* genome then the Ena/VASP family of proteins, where Ena functions in place of three mammalian homologs. It is interesting to speculate that the mammalian homologs could be the result of a gene duplication event of an ancestral gene that is similar to *naus.*

### Naus's lamellipodial enrichment is Cortactin-dependent

We used TIRF microscopy in combination with RNAi treatments and point mutations and determined that Naus’s lamellipodial enrichment is Cortactin-dependent. Using two different RNAi sequences targeting Cortactin, we found that depletion of Cortactin leads to a loss of Naus localization at actin structures in the lamellipodia. This differs from the mammalian counterpart in which re-localization of Cortactin by glutamate stimulation in neurons does not lead to CTTNBP2 redistribution. We observed a similar loss in localization upon expression of a point mutant where the proline residues in the conserved proline patch (Fig. S1) were mutated to alanine (amino acid positions 563–567). Thus Naus, like its mammalian counterparts, uses this same conserved region for its association with Cortactin, though their mechanisms of localization differ. Along with abolishing the interaction with Cortactin in COS cells, a similar proline mutant of rat CTTNBP2 failed to rescue the decrease in dendritic spine density following depletion of endogenous CTTNBP2 (Chen et al., 2012), arguing that not only is this association important for proper localization, but it is also needed for proper function.

### Naus and Cortactin regulate one another's dynamics in a reciprocal manner

Our kinetic studies of Naus and Cortactin reveal a mutual relationship between the two proteins. By both FRAP and PARF, we found that following Cortactin RNAi, Naus was no longer anchored to the actin cytoskeleton. This data supports our localization studies and further implicates Cortactin's role in recruiting Naus to the cytoskeleton. Cortactin is incorporated throughout the lamellipodia where it likely targets nascent actin branch junctions having a 300-fold increased affinity junctions over the sides of actin filaments (Helgeson and Nolen, 2013). Interestingly, using *in vitro* single-molecule imaging, Helgeson and Nolen observed that Cortactin's average lifetime at existing branch junctions during active branching was 29.5 s (Helgeson and Nolen, 2013), while the lifetime of branches *in vitro* has been observed to be between 8 and 27 min (Mahaffy and Pollard, 2006; Martin et al., 2006). Our kinetic data (Fig. 4) suggests that Naus may function to retain Cortactin throughout the lamellipodia, likely at branch junctions given Cortactin's high affinity for these sites. While this increase in the time at junctions may not be on par with the reported lifetimes *in vitro*, lamellipodial actin undergoes treadmilling. Thus, this delay in the dissociation of Cortactin as a result of its association with Naus may be more consequential *in vivo*. Delays in Cortactin's dissociation could also lead to a decrease in the number of new branches given its ability to function as a type II NPF.

### Naus regulates branch density, actin-retrograde flow rates and lamellipodial protrusion

Quantitative fluorescence speckle microscopy revealed that depletion of Naus leads to a decrease in actin retrograde flow speeds within the lamellipodia (Fig. 3). This decrease in retrograde flow may be the result of an overall decrease in Arp2/3 nucleation and branch junction stabilization. Three critical observations led us to this hypothesis. Firstly, our kinetic data indicates Cortactin more readily dissociates from the actin cytoskeleton following Naus depletion. Secondly, we found that RNAi depletion of Naus led to an increase the number of long unbranched actin filaments by platinum replica electron microscopy (Fig. 6). Finally, quantitative imaging suggests that depletion of either Naus or Cortactin leads to a decrease in fluorescence intensity of the Arp2/3 complex throughout the lamellipodia (Fig. 7). Thus, in the absence of Naus, Cortactin could be undergoing a cycle of precocious dissociation from branch junctions leading to less activation of the Arp2/3 complex and a decrease in the stability of branched junctions resulting in an overall reduction in incorporation of the Arp2/3 complex in the lamellipodia. In an *in vitro* de-branching assay, Cai and colleagues observed an increase in the density of branches following a second-wave perfusion of Cortactin. Similarly, in a centrifugation assay, Cortactin was able to overcome the debranching catalyzed by Coronin 1B and increase the amount of amount of Arp2/3 associated with F-actin (Cai et al., 2007). This group also found that Cortactin depletion led to a decrease in actin retrograde flow (Cai et al., 2007), similar to what we observed in S2R+ cells following Naus depletion (Fig. 5). Our results place Naus upstream of Cortactin and thus upstream of Arp2/3 mediated branch nucleation.

Not surprisingly, the changes to the actin architecture and the amount of Arp2/3 incorporated into the lamellipodia also lead to changes in lamellipodial dynamics. These longer less-branched actin filaments decreased the speed of cell migration and the directionality of this migration, but increased the amplitude of lamellipodial protrusions and increased lamellipodial persistence (Fig. 8). Interestingly, depletion of Cortactin also led to a decrease in random cell motility, however these cells had less persistent lamellipodial protrusions suggesting nuanced differences between the loss of Cortactin and the loss of Naus to actin dynamics (Bryce et al., 2005). These differences may very well lie in function of Naus to stabilize Cortactin retaining it in the lamellipodia. Naus depletion appears to differ somewhat from the depletion of the branch destabilizer Coronin 1B as well. The depletion of Coronin 1B leads to a more densely-branched actin network and a decrease in retrograde flow rates. Coronin 1B depletion also leads to an increase in the speed of lamellipodial protrusion while reducing lamellipodial persistence (Cai et al., 2007; de Hostos et al., 1993; Krause and Gautreau, 2014). Thus, Naus's role in fine-tuning the lamellipodia is distinct from that of both Cortactin and Coronin 1B.

Many of these conclusions have been drawn from experiments where Cortactin levels have been reduced by RNAi depletion, however, results from Lai and colleagues using genetically Cortactin-null fibroblasts tell a slightly different story. These researchers found very little differences in the lamellipodial dynamics of Cortactin-null fibroblasts as compared to wild-type fibroblasts, but did observe slightly slower rates of randomly migrating cells and the rates of wound closure in scratch-wound assays. Furthermore, the rates of actin incorporation into the lamellipodia were slightly reduced (Lai et al., 2009). Moreover, Sung and colleagues found that many of the Cortactin depletion phenotypes were the result of Cortactin dependent secretion of fibronectin (Sung et al., 2011; Schoor et al., 2018). Of note, S2R+ cells lack the appropriate integrin pairs to adhere to ECM (Bunch and Brower, 1992; Ribeiro et al., 2014) and are plated on a lectin where they are likely not engaged in integrin signaling or secretion. Some of these seemingly contradictory results may point to phenotypes that arise from differences in dosage, cell type or long term compensatory mechanisms. It is clear from these findings that the exact role of Cortactin in actin dynamics, lamellipodial formation and cell migration has yet to be clearly defined.

### Naus plays a role in regulating neuronal morphology

This role in regulating actin dynamics also plays out in determining the morphology of neurons. Using fly genetics, we depleted Naus in neuroblasts, which differentiate into neurons in culture. We found that depletion of Naus led to a decrease in the number of neuronal processes made in comparison to wild-type neurons (Fig. S8). Similarly, depletion of CTTNBP2 also led to a decrease in neuronal arborization as well as a decrease in the density of dendritic spines (Chen and Hsueh, 2012; Chen et al., 2012; Shih et al., 2014). However, CTTNBP2 also promotes microtubule stability, thus its role in promoting neuronal arborization may have diverged from its role in regulating Cortactin dynamics (Shih et al., 2014). Interestingly, we did not observe wild-type Naus associating with microtubules and it was only upon expression of the alanine mutant (Naus-AAAIA, Fig. S2D), albeit on a rare occasion, did we observed co-localization with microtubules. Understanding the differences between Naus and CTTNBP2 will likely be the focus of future studies, particularly if they both contribute to the morphology of neurons in distinct ways despite being closely related.

### Working model for Naus's role in branch nucleation, stabilization and the fine-tuning of the lamellipodia

Given the observations detailed here, we propose a model wherein Naus acts through the stabilization of Cortactin at Arp2/3 generated branches to regulate their dynamics (Fig. S9). By stabilizing Cortactin, Naus inhibits its precocious dissociation while preventing debranching. Without Naus, Cortactin more freely dissociates from Arp2/3 generated branches leading to the destabilization of branch junctions and an overall decrease in actin branch density throughout the lamellipodia. This decreased density leads to larger scale cellular changes, such as reduced speeds in cell migration (Fig. 8) and a decrease in the number and arborization of neuronal branches observed in this study (Fig. S8). In a similar manner, Cortactin maintains Naus at the lamellipodia and when Cortactin is absent, Naus loses this enrichment and, in extremely rare cases, relocalizes to other structures such as microtubules. Collectively, both Naus and Cortactin act in concert to ensure the appropriate spatial and temporal regulation of lamellipodial actin dynamics.

## MATERIALS AND METHODS

### Cell culture and RNAi

*Drosophila* S2R+ cell culture and RNAi were performed as described in Rogers and Rogers (2008) and Applewhite et al. (2016). Briefly, S2R+ (Drosophila Genomics Resource Center) cells were cultured in Shields and Sanger media (Sigma-Aldrich) supplemented with 100× antibiotic-antimycotic (Thermo Fisher Scientific) and 10% fetal bovine serum (Thermo Fisher Scientific) maintained at 25°C. RNAi was administered in six-well plates by treating cells (approximately 50% confluent) with 10 μg of double-stranded RNA (dsRNA) in 1 ml of medium each day for 7 days. Control RNAi was made from dsDNA amplified from pBlueScript vector with no known homology to the *Drosophila* genome. For all other dsRNA targets please see Table S1 for primer sequences.

*Drosophila* ML-DmD25c2 (D25 cells, Drosophila Genomics Resource Center) were maintained as described in Currie and Rogers (2011). Briefly, D25 cells were cultured in Schneider's media (Thermo Fisher Scientific) supplemented with 100× antibiotic-antimycotic (Thermo Fisher Scientific), 10% fetal bovine serum (FBS, Thermo Fisher Scientific) and 10 μg/ml insulin (Thermo Fisher Scientific). RNAi regimen was the same as described for S2R+ cells (see above).

*Drosophila* primary neuroblasts were harvested and cultured as described in Lu et al. (2013). Briefly, the brains of third instar larvae were dissected in Schneider's media supplemented with 20% FBS and then enzymatically dissociated with liberase (Roche, Basel, Switzerland) at a final concentration of 0.20–0.25 mg/ml in Modified Dissecting Saline (137 mM NaCl, 5.4 mM KCl, 0.17 NaH_2_PO_4_ 0.22 mM HKPO_4_ 3.3 mM Glucose, 43.8 mM Sucrose, 9.9 mM Hepes, pH 7.5). The Modified Dissecting Solution was replaced with Schneider's media supplemented with 20% FBS and neuroblasts were plated on ECM harvested from the D25 cells (see Currie and Rogers, 2011) and allowed to differentiate for 24 h at 25°C.

### Molecular biology

The cDNA clones for Naus (CG10915) and Cortactin (CG3637) were obtained from the Drosophila Genomics Resource Center and were cloned into pMT or pIZ (Invitrogen) vectors following standard PCR procedures. Naus's conserved Cortactin binding motif (amino acid positions 563–567) were mutated to alanine by site-directed mutagenesis.

### Immunofluorescence and live-cell imaging

Cells were prepared for immunofluorescence and live-cell imaging as described in Applewhite et al. (2016). S2R+ cells were plated on concanavalin A-treated coverslips attached to laser cut 35 mm tissue culture dishes with UV-curable adhesive (Norland Products, Cranbury, USA) in Shields and Sanger media supplemented with 10% FBS and 100× antibiotic-antimycotic for both fixed and live-cell imaging. D25 cells were plated on glass-bottom dishes (described above) treated with ECM harvested from the cells as described in Currie and Rogers (2011). Antibodies used in this study include anti-Myc 9E10, anti-Futsch, anti-alpha and anti-beta tubulin (all from Developmental Hybridoma Bank) diluted 1:200 in a 5% solution of normal goat serum (Sigma-Aldrich) and phosphate-buffered solution with 0.1% Triton x-100 (PBST) (Sigma-Aldrich). Secondary antibodies (Alexa-488 and Alexa 594; Jackson ImmunoResearch) and phalloidin (Alexa-488 and Alexa-594; Thermo Fisher Scientific) were used at final dilution of 1:100 in PBST. Hoechst (Thermo Fisher Scientific) was diluted 1:10,000 in PBST. All transfections were carried out using using FuGENE HD (Promega). Expression of pMT vectors was achieved with 250–500 μM final concentration of copper sulfate unless noted otherwise. Cells were fixed using a 10% solution of Paraformaldehyde (Electron Microscopy Sciences, Hatfield, USA) and PEM buffer (100 mM Pipes, 1 mM EGTA, 1 mM MgCl_2_). Fixed cells were mounted using Dako anti-fade mounting media (Agilent, Santa Clara, USA). All imaging was performed on a TIRF system mounted on an inverted microscope (Ti-E, Nikon, Tokyo, Japan) using a 100×/1.49NA oil immersion TIRF objective driven by Nikon Elements software unless noted otherwise. Images were captured using an Orca-Flash 4.0 (Hamamatsu, Japan) and were processed for brightness and contrast using ImageJ before analysis.

### Immunoblotting

S2R+ cells were treated with RNAi targeting Naus or Cortactin for 7 days. On the fifth day of treatment cells were transfected with either pMT-EGFP-Naus or pMT-myc-Cortactin and induced to expression the transgene overnight with 600 µM CuSO_4_. Whole-cell lysate from the cells was then collected by re-suspending cell pellets with SDS-sample buffer following by boiling for 5 min. The efficiency of RNAi was determined by western blotting the lysates where equal protein amounts were loaded. Antibodies used in this study were diluted in standard 5% milk Tris- buffered solution plus 0.1% Tween, pH 7.4 (TBST) solution and include anti-GFP (1: 5000, Abcam), anti-myc 9E10 (1:200, Drosophila Hybridoma Bank), anti-beta tubulin 12G10 (1:200, Drosophila Hybridoma Bank) and anti-mouse HRP (1:5000, Cell Signaling).

### Co-localization analysis

Co-localization was analyzed by line-scan analysis and Mander's coefficient analysis. For line-scan analysis, a 10 μm line was drawn from the cell edge inward and fluorescence intensity was measured. These values were normalized and then averaged for all cells within that condition. Mander's coefficient analysis was performed using the Just Another Colocalization Program (JACoP) plugin for ImageJ (Bolte and Cordelières, 2006). Briefly, intensity thresholds were manually set for both fluorescence channels and then the fraction of overlap was calculated in each direction.

### Neuroblast analysis

Neuroblasts were analyzed using the Simple Neurite Tracer and Sholl Analysis plugins in ImageJ (Ferreira et al., 2014; Longair et al., 2011). For Sholl Analysis, neuroblasts were converted to a threshold image. Following this, a line from the center of the soma to past the further branch tip was drawn to define the space for analysis. The radius for analysis was set to 2 μm concentric circles and the number of intersections per radius was calculated. Similarly, the max Sholl radius was then extracted by the maximum radius at which the number of intersections was greater than zero. For analysis of average branch length and number of branches using Simple Neurite Tracer, a line skeleton of the neuron image was manually drawn and these values were then calculated using the plugin.

### Permeabilization activated reduction in fluorescence

PARF was performed as described in Singh et al. (2016). Briefly, cells were prepared for live imaging as described above. Time-lapse images were captured with constant exposure at a rate of one frame every 2 s. After 40 s (20 frames), digitonin (25 μM final concentration) was added. Cells from the same dish were imaged under the same conditions but without digitonin treatment for use as a photofading due to acquisition (PDA) control. Analysis was performed using ImageJ and GraphPad Prism 6. The area of each region of interest (ROI) was held constant. An ellipse of the background of each movie (control and digitonin treatment) was selected and intensity density was determined for the background in each frame. PDA was determined as previously described in Applewhite et al. (2007). The intensity density was determined for a lamellipodial ROI in the control (no digitonin treatment) movie for each plate. The background intensity was subtracted and change in fluorescence was fit to a one-phase exponential decay of the following general equation where I is intensity, k is the photofading factor, e is Euler’s constant, t is time in seconds and I_o_ is the initial intensity: 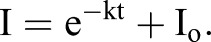
The intensity density of a lamellipodial ROI for digitonin cells was obtained in the same manner. Background intensity was subtracted and the intensity density was then multiplied by e^kt^. The intensity was normalized for each cell and the data were averaged for each condition. To compare the half-life between conditions for statistical significance, the normalized fluorescence for each cell was fit to a two-phase exponential decay and t_1/2_ was determined. These half-life values were averaged in each condition and compared using a two-tailed Student's *t*-test.

### Fluorescence recovery after photobleaching

FRAP was performed using a Zeiss LSM880 laser-scanning confocal microscope (Jena, Germany). Cells were prepared for live imaging as described above. Time-lapse images were captured every 1.34 s. After 50 cycles (65.6 s), selected regions of the cell were bleached (five iterations) and the intensity was recorded. Intensity was also recorded for a non-bleached region and a background region of the same size was used for PDA controls. FRAP analysis was performed as described in Applewhite et al. (2007). The fluorescence intensities in the bleached zone in each frame were measured. The background was subtracted and the intensity was corrected for photofading as described above. The intensity was normalized for each ROI. This corrected intensity was fit to a one-phase association. The half-life of recovery was calculated as ln2 k^−1^. Values were compared using a two-tailed Student's *t*-test.

### Quantitative imaging and analysis

For quantification of F-actin in the lamellipodia, RNAi-treated S2R+ cells were prepared in parallel and fixed using a 10% solution of Paraformaldehyde (Electron Microscopy Sciences, Hatfield, PA) and PEM buffer (100 mM Pipes, 1 mM EGTA, 1 mM MgCl_2_). Cells were stained for F-actin using phalloidin (Alexa-488, Thermo Fisher Scientific) at final dilution of 1:100 in PBST. Fixed cells were mounted using Dako anti-fade mounting media (Agilent). All imaging was performed on a total internal reflection fluorescence (TIRF) system mounted on an inverted microscope (Ti-E, Nikon) using a 100×/1.49NA oil immersion TIRF objective driven by Nikon Elements software unless noted otherwise and images were captured using an Orca-Flash 4.0 (Hamamatsu). F-actin intensity was analyzed by line-scan analysis and ROI. For line-scan analysis, a 10 µm line was drawn from the cell edge inward along which the fluorescence intensity was measured. These values were background subtracted, normalized and then averaged for all cells within that condition. To measure the mean fluorescence intensity of the lamellipodia an ROI of the cell perimeter was made and the fluorescence intensity was measured from the cell perimeter inward 2.7 µm encompassing the lamellipodia. The mean intensity along this perimeter was measured and plotted for each condition.

For quantification of Arp3 in the lamellipodia, RNAi-treated S2R+ cells were prepared in parallel and simultaneously fixed in a solution of PEM buffer (100 mM Pipes, 1 mM EGTA, 1 mM MgCl_2_) supplemented with 0.5% Triton-X-100 and 0.25% glutaraldehyde (EM grade from Electron Microscopy Sciences) for approximately 2 min followed by a 15-min fixation in 2% glutaraldehyde in 0.1 M sodium cacodylate, pH 7.3. Following a rinse with PBD, the cells were then incubated overnight with anti-Arp3 antibody (A5979, Sigma-Aldrich) diluted 1:400 in 5% normal goat serum (Sigma-Aldrich) in PBST. The cells were rinse again with PBS and then incubated with anti-mouse Alexa-488 (Jackson ImmunoResearch) and Alexa-595 (Thermo Fisher Scientific) diluted 1:100 in PBST and mounted with anti-fade mounting media (Dako). All imaging was performed on a total internal reflection fluorescence (TIRF) system mounted on an inverted microscope (Ti-E, Nikon) using a 100×/1.49NA oil immersion TIRF objective driven by Nikon Elements software unless noted otherwise and images were captured using an Orca-Flash 4.0 (Hamamatsu). The fluorescence of Arp3 was measured along lines 1.75 µm in length encompassing 0.875 µm of the background and 0.875 µm of the lamellipodia. 8–10 cells were measured per condition with three independent lamellipodia measurements taken. These values were background subtracted, normalized and then averaged for all cells within that condition. The mean raw fluorescence intensities from these measurements were also recorded (Fig. 7F,H) with the background measurements excluded.

### Quantitative fluorescence speckled microscopy

RNAi treatment and transfection was performed as described above. Following transfection, cells were induced with 30 μM copper sulfate and incubated overnight. Live cell movies were obtained at 200 ms exposure in 2 s intervals for 2 min. The resulting movies were analyzed using a previously described Quantitative Fluorescent Speckle Microscopy (QFSM) software in MATLAB (Mendoza et al., 2012). Images were acquired at a rate of 30 frames per minute (130 nm per pixel, NA=1.4, 16 bit images). Full cell masks were generated using automatic thresholding (MinMax setting). Flow analysis was performed using the flow tracking setting with a six-frame integration window. For cell-wise quantification of lamellipodial flow rates, masks of lamellipodial regions for each cell were generated and the average actin flow rate was calculated for the first 30 s of each movie (*n*=27−35 cells per group). To compare the overall fluorescence of the cells used in QFSM an ROI encompassing the cell perimeter was made and the mean fluorescence intensity of this ROI was recorded for each cell.

### Platinum replica electron microscopy

Sample preparation for platinum replica electron microscopy was performed as previously described in Svitkina (2016). Briefly, cells were extracted in Extraction buffer [1% Triton X-100, 2% PEG (MW 35 kDa) in PEM buffer supplemented 2 µM phalloidin], washed with PBS and then fixed with 2% glutaraldehyde (EM grade from Electron Microscopy Sciences) in 0.1 M sodium cacodylate, pH 7.3. Fixed cells were then treated with 0.1% tannic acid and 0.2% uranyl acetate in water, critical-point dried and coated with platinum and carbon. They were then transferred to EM grids for imaging. All EM measurements were made on images of 30,000× resolution (high magnification images Fig. 6A–H). As distinct branch sites are difficult to determine from the largely dense network, especially in the case of control images, we measured the distance between branch points or filament intersections in each condition to get a rough estimate of the branching in the lamellipodial network. Measurements between branches or filament intersections were made by drawing a segmented line from one filament branch or crossing to the next, for those that could be discernibly determined as the same filament or same filament bundle. At least 40 measurements were made per image, across five separate images for each condition (*n*>200 per condition).

### Random cell migration assay and kymography

D25 cells were plated at a subconfluent density on ECM-coated glass-bottom dishes and allowed to attach overnight. Cells were imaged every 5 min for 6 h by phase-contrast microscopy using 40×/0.75NA objective. Individual cells were manually tracked using Manual Tracker (ImageJ). Cell directionality was calculated as a ratio of the direct distance between start and end points (D) to the total path length taken by the cells (T). To measure the rates of lamellipodial protrusion, retraction, persistence, frequency and amplitude, kymographs were made using the Multi Kymograph ImageJ plugin from phase-contrast movies acquired every 2 s for 10 min. Kymographs were generated from phase-contrast movies of migrating D25 cells acquired every 2 s for 10 min. A line approximately 16 µm in width was drawn from the center of the cell to a few microns beyond the cell periphery. Following the protocol established by Hinz et al. (1999), these kymographs were used to extract the lamellipodial protrusion parameters. All statistics for this manuscript were performed using GraphPad Prism 6 unless otherwise noted.

## Supplementary Material

Supplementary information
