# Supplementary material for: The Drosophila protein, Nausicaa, regulates lamellipodial actin dynamics in a Cortactin-dependent manner
Source: Biol Open. 2019 Jun 4;8(6):bio038232. doi: 10.1242/bio.038232 (PMC6602326; doi:10.1242/bio.038232)
Supplement: Supplementary information [file biolopen-8-038232-s1.pdf]

Table S1. Forward and reverse primer sequences for production of cloning and dsDNA (for dsRNA production) in this study.

Gene Name	CG Number	Use	Forward Primer (5'-3')	Reverse Primer (5'-3')	Reference
p20 of Arp2/3 complex	CG5972	dsDNA for RNAi	CAGACAACAACCCGACACC	CAGTTTCATTTCGCTGATCTCC	(Rogers et al., 2003)
Cortactin	CG3637	dsDNA for RNAi	ATCAGAACGCAGGATACGGA	GAGACTCGTGCTTCTCCACC	Harvard Fly RNAi (Flockhart et al., 2006)
Cortactin	CG3637	dsDNA for RNAi	AAGTCGGCTGTGGGTCATGA	CTGGCTTGACCTTGTGGTCC	This study
Cortactin	CG3637	pMT-mCherry-Cortactin	ATGTGGAAGGCAAGTGCCGGTATGTGGAAGGCAAGTGCCGGT	TTATGAGTTCTGTCCCACCACCTGC	This study
Cortactin	CG3637	pMT-myc-Cortactin	ATGGAGCAAAAACTCATTAGCGAAGAAGACTTAATGTGGAAGGCAAGTGCCGGT	TTATGAGTTCTGTCCCACCACCTGC	This study
Nausicaa	CG10915	dsDNA for RNAi	CACGAGTTCCAGACCATGAA	CCCTCTGCTTGCTAACCATC	Harvard Fly RNAi (Flockhart et al., 2006)
Nausicaa (5'UTR)	CG10915	dsDNA for RNAi	CTTTTTCACGGGCAACAAAT	CGGTTTCATGGTCTGGAACT	Harvard Fly RNAi (Flockhart et al., 2006)
Nausicaa	CG10915	pIZ-Naus-EGFP	CACCATGGAGCAGAACTCGAACAGCAGCGTT	ATTCTGCTTCGCCGTGCCCGC	This study

Biology Open • Supplementary information

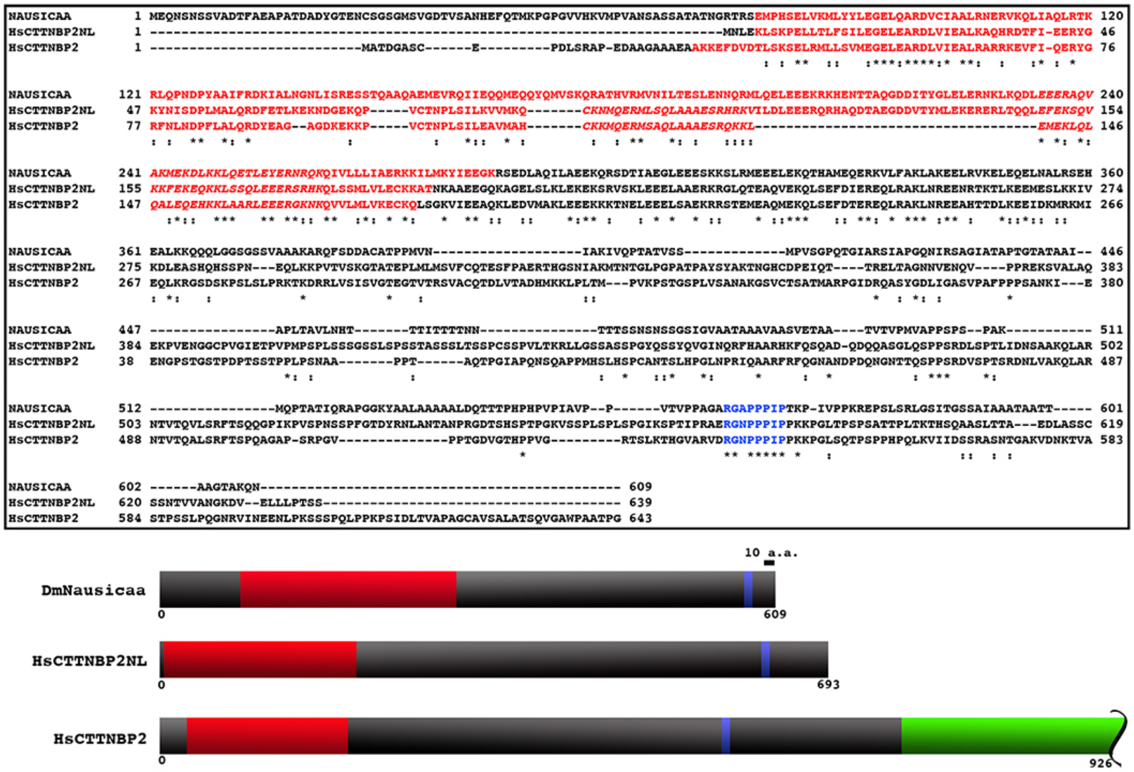

Fig. S1. Sequence Alignment of *Drosophila* Nausicaa with *Homo sapien* Cortactin binding protein 2 (CTTNBP2) and Cortactin binding protein 2 N-terminal like (CTTNBP2NL). (Top) A multiple sequence alignment made using Clustal Omega comparing *Drosophila melanogaster (Dm)* Nausicaa (CG10915) and *Homo sapien (Hs)* CTTNBP2NL, and CTTNBP2. Nausicaa shares approximately 30% identity with *Hs* CTTNBP2NL and 28% identity with *Hs* CTTNBP2. Shown in red is the conserved Cortactin Binding Protein-2 (CortBP2) domain, in red italics a predicted a coiled-coil domain, and in blue the poly-proline Cortactin binding motif. Asterisks indicate identical amino acids while colons indicate similar amino acids. Note that only the first 643 amino acids of *H.s.* CTTNBP2 are shown. (Bottom) Diagram of *Dm* Nausicaa, *Hs* CTTNBP2NL and *Hs* CTTNBP2 drawn to scale. In red is the conserved CortBP2 domain, in blue the Cortactin binding motif, and in green the COOH-terminus of CTTNBP2. Note that only the first 926 amino acids of CTTNBP2 are shown.

Biology Open • Supplementary information

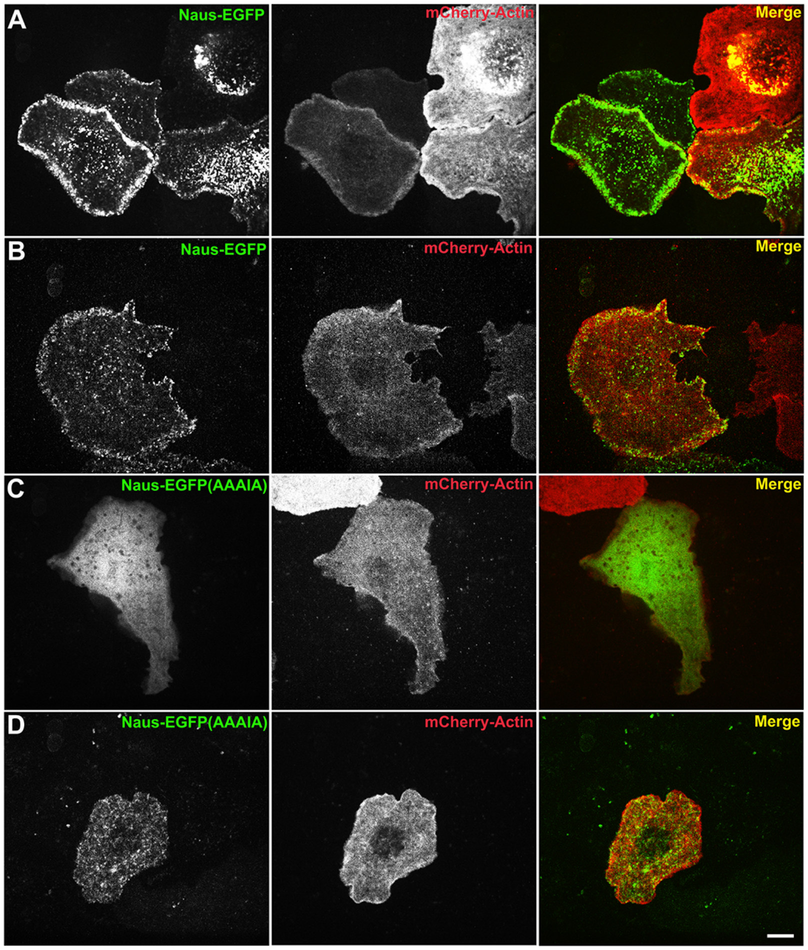

Fig. S2. Wild-type Nausicaa is enriched the circumferential lamellipodial of *Drosophila* S2R+ cells. S2R+ cells imaged by spinning disk confocal microscopy co-expressing (A-B) EGFP-tagged wild-type Naus or (C-D) AAAIA mutant of Naus (green, left panels) with mCherry-Actin (red, middle panels) imaged by spinning disk confocal microscopy. Right panels are the merge of the two images. Cells pictured A and C expressed higher levels of the EGFP-tagged protein then the cells pictured in B-D. Scale bar = 10 μm.

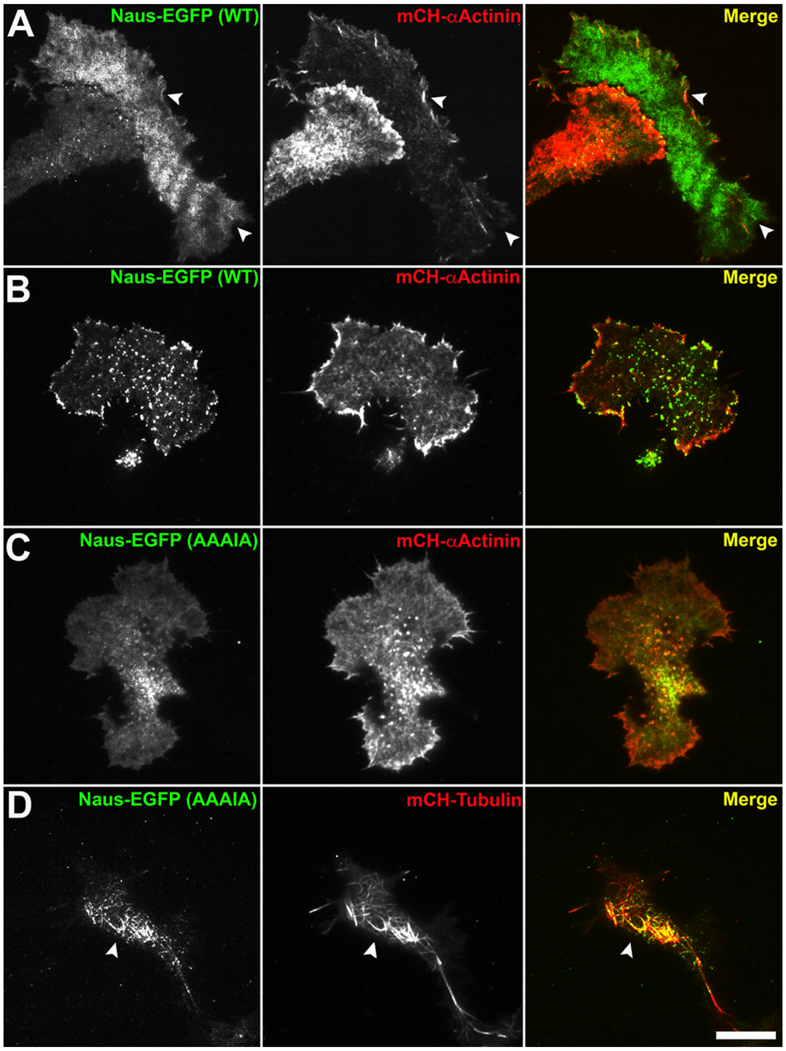

Fig. S3. Nausicaa weakly localizes to α-Actinin bundles in D25c2 cells through a proline rich motif, while mutant Naus localizes to microtubules. (A-D) Representative live-cell images of D25 cells imaged by TIRF microscopy co-transfected with mCherry-αActinin (A-C), or mCherry-Tubulin (D) (middle panels, red in merged images) and Naus-EGFP WT (A - B) or Naus AAAIA mutant (C-D) (left panels, green in merged images). (B) White arrowheads indicate bundled-actin structures containing both mCherry-α-Actinin and Naus-EGFP. (D) White arrowheads indicate where Naus-EGFP (AAAIA) appears to localize to microtubules in D25 cells expressing mCherry Tubulin (red in merged). Scale bar = 10 μm.

Biology Open • Supplementary information

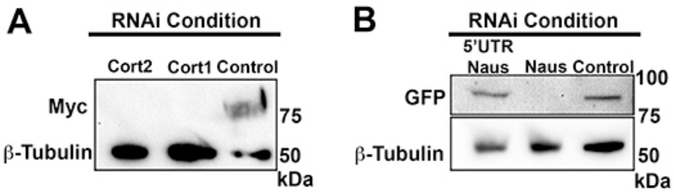

Fig. S4. RNAi depletion of Nausicaa and Cortactin. S2R+ cells were treated with RNAi for seven days, transiently transfected with metallothionein inducible transgenes on the fifth day and induced for 12 hours on day six to seven with 600 μM $CuSO_4$. (A) S2R+ cells were treated with two independent RNAi sequences targeting Cortactin or control RNAi and transfected with Myc-tagged Cortactin. Whole cell lysate was collected and immunoblotted for anti-Myc and anti-β-tubulin. (B) S2R+ cells treated with RNAi targeting the 5'UTR of Naus, the coding sequence of Naus (Naus), or control for seven days and transfected with GFP-tagged Naus. Whole cell lysate was harvested and immunoblotted for anti-GFP and anti-β-tubulin.

Biology Open • Supplementary information

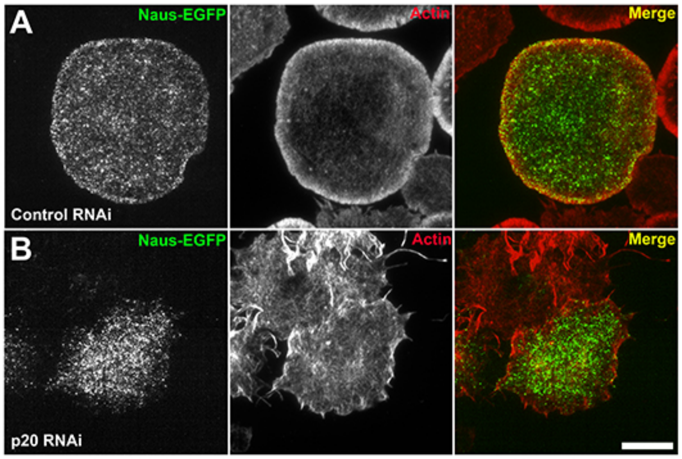

Fig. S5. Nausicaa's lamellipodial localization is dependent on Arp2/3 complex. Fixed S2R+ cells imaged by TIRF microscopy expressing Naus-EGFP (left panels, green in merged images) and treated with either control (A) or p20 (B) RNAi. Cells are stained for F-actin with phalloidin (middle panel, red in merged images). Scale bar = 10 μm.

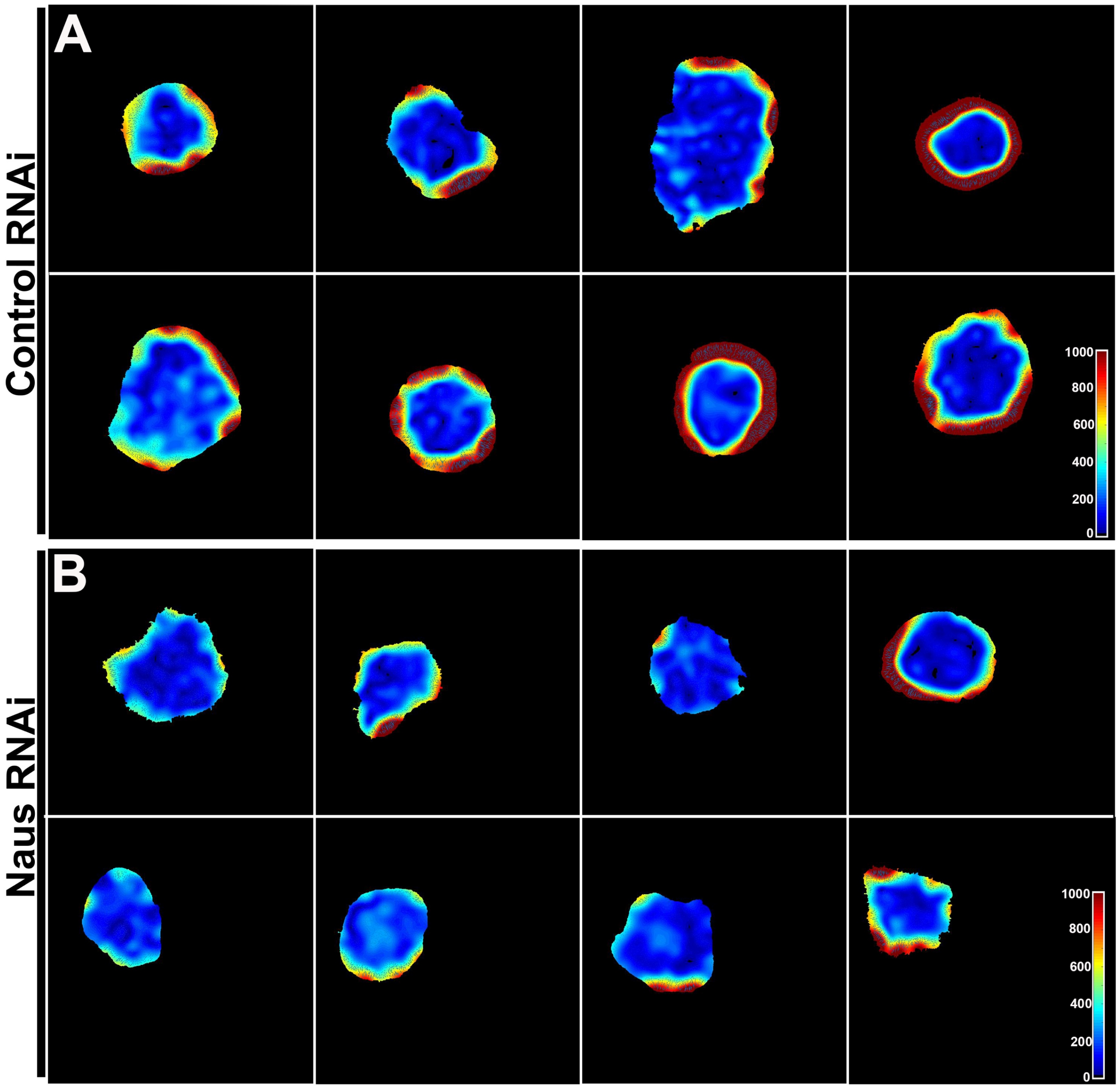

Fig. S6. Representative heat maps from QFSM analysis. Heat map of the speed of actin retrograde flow in S2R+ cells following (A) control or (B) Naus RNAi.

Biology Open • Supplementary information

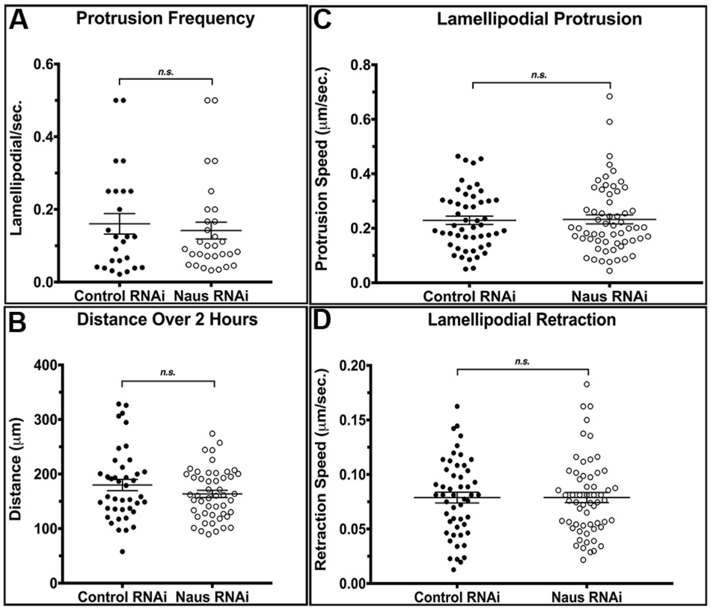

Fig. S7. Nausicaa regulates specific lamellipodial parameters. Quantification of lamellipodial dynamics such as frequency of protrusions (A), the distance the cells traveled over two hours (B) the speed of protrusions (C), and the speed of retractions (D) n.s.= not signification (two-tailed Student's t-test). N= 51 control cells and 59 Naus RNAi treated cells (A,C,D) or N= 44 control cells and 50 Naus RNAi treated cells (B).

Biology Open • Supplementary information

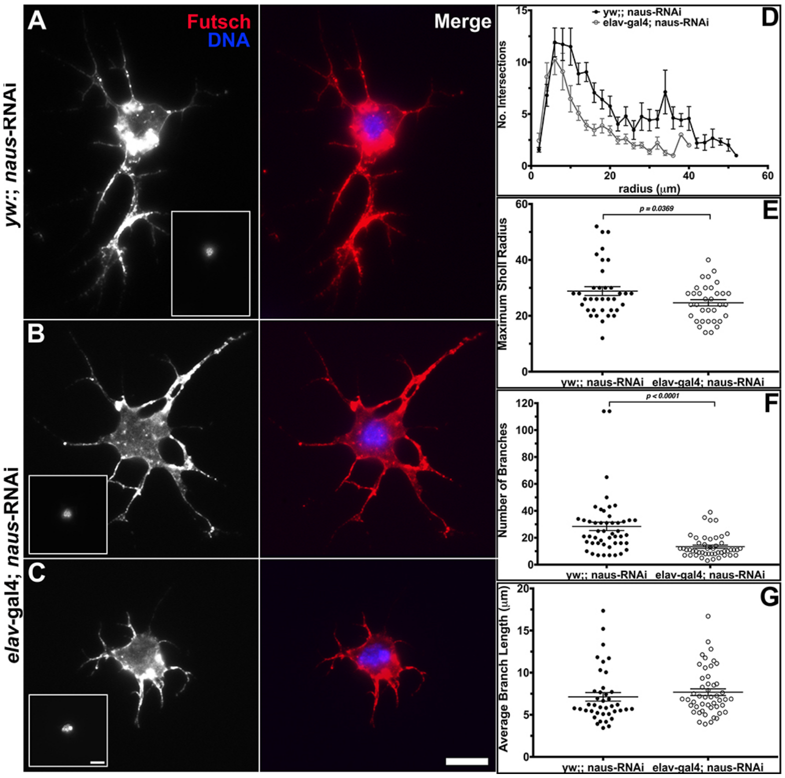

Fig. S8. Nausicaa regulates neuronal morphology in 3rd instar larvae neurons. (A-C) Primary neuroblasts from 3rd instar larvae were harvested and allowed to differentiate for 24 hours in culture. They were then fixed and stained for microtubules (anti-Futsch, red in merged images) and DNA (Hoechst, inset and in blue in merged images). (A) Control neurons from yw;; *naus*-RNAi flies. (B - C) Neurons from flies expressing *naus*-RNAi driven by *elav*-gal4. Scale bars = 10 μm.(D) Quantification of the Sholl profile, which measures the number of intersections a neuron makes, (error bars denote S.E.M.). (E) Quantification of the maximum Sholl radius, which measures the number of branch intersections from concentric circles in Naus RNAi neurons (open black circles) and control neurons (black circles) (*p-value = 0.0369, Student's t-test, control: n = 36 cells, Naus RNAi: n = 34 cells). (F - G) 2D skeletons of neurons were manually drawn and analyzed ImageJ Simple Neurite Tracer for (F) number of branches and (G) the average branch length. (F) for Naus depleted neurons (open black circles) and control neurons (black circles) prepared in parallel (***p-value <0.0001, Student's t-test, Control: n = 49 cells, Naus RNAi: n = 48 cells). Error bars = S.E.M.

Biology Open • Supplementary information

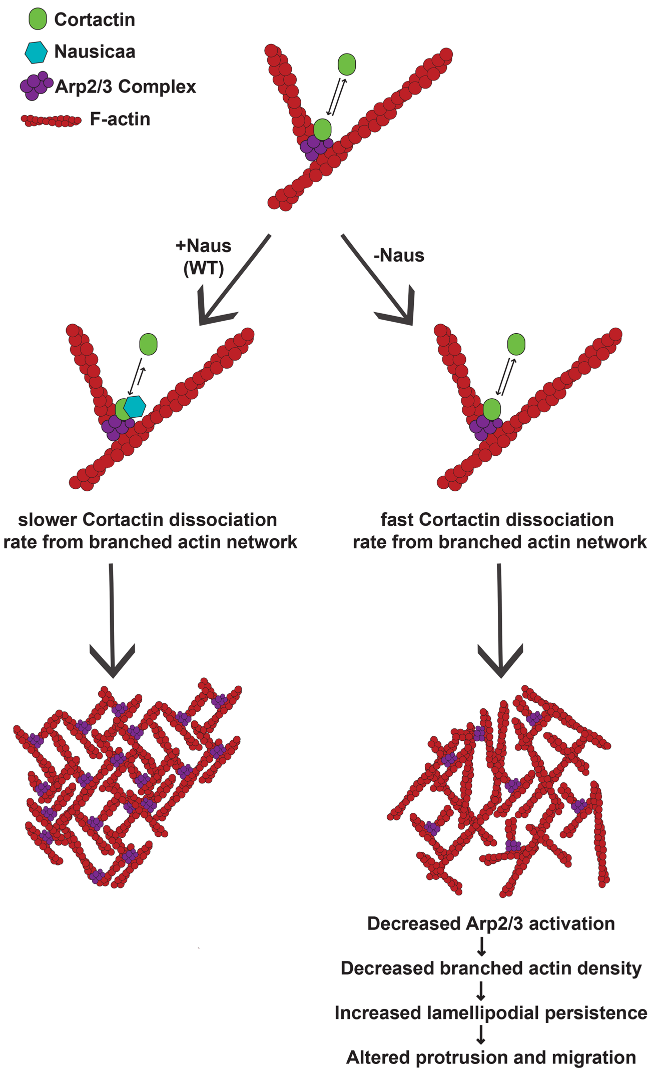

Fig. S9. Proposed model of Nausicaa's role in the lamellipodia. Nausicaa works to stabilize Cortactin at Arp2/3 generated branches in order to appropriately regulate branch density of the lamellipodia. Nausicaa's interaction with Cortactin maintains Cortactin on branched actin networks and stabilizes these junctions. In the absence of Nausicaa, Cortactin has a fast off-rate from Arp2/3 branches (Helgeson and Nolen, 2013). Cortactin more freely diffuses leading to a decrease in the activation of the Arp2/3 complex and a loss of branch stability. This increased filament length leads to larger lamellipodial protrusions and downstream alterations to migration and morphology.

Movies.

All time-lapsed images are played at a rate of 7 frames per second. All images were acquired by TIFR microscopy unless otherwise noted.

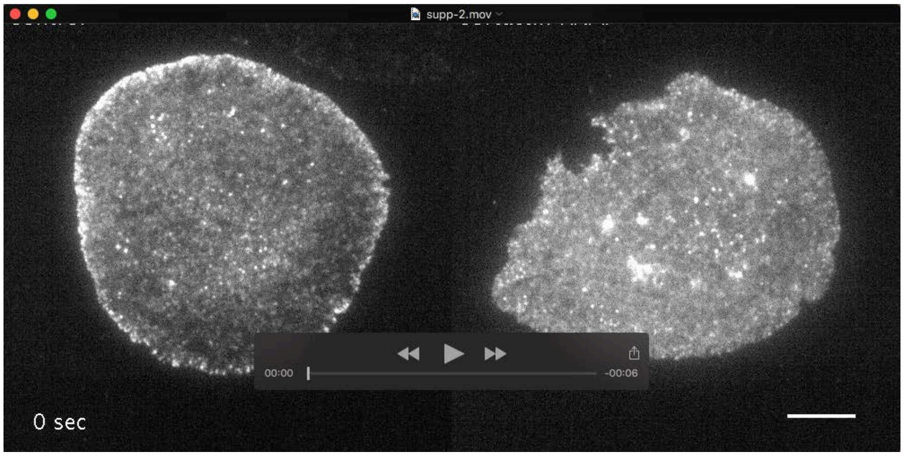

Movie 1. Lamellipodial localization of Naus is Cortactin-dependent.

S2R+ cells expressing EGFP-tagged Naus following control RNAi (right) or Cortactin RNAi (left). Image sequence was acquired at 3 second intervals.

Biology Open • Supplementary information

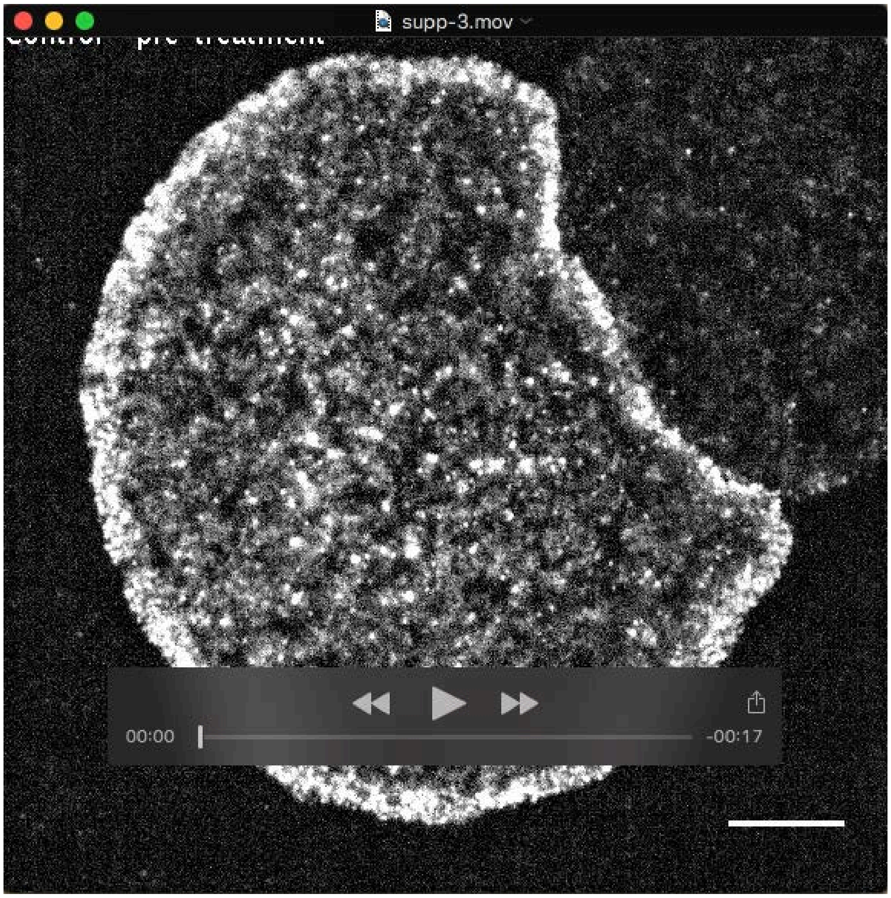

Movie 2. PARF of Naus following treatment with control RNAi. An S2R+ cell expressing EGFP-tagged Naus following control RNAi. After 40 seconds of imaging, the cell was permeabilized with 25 μM digitonin. Image sequence was acquired at 2 second intervals.

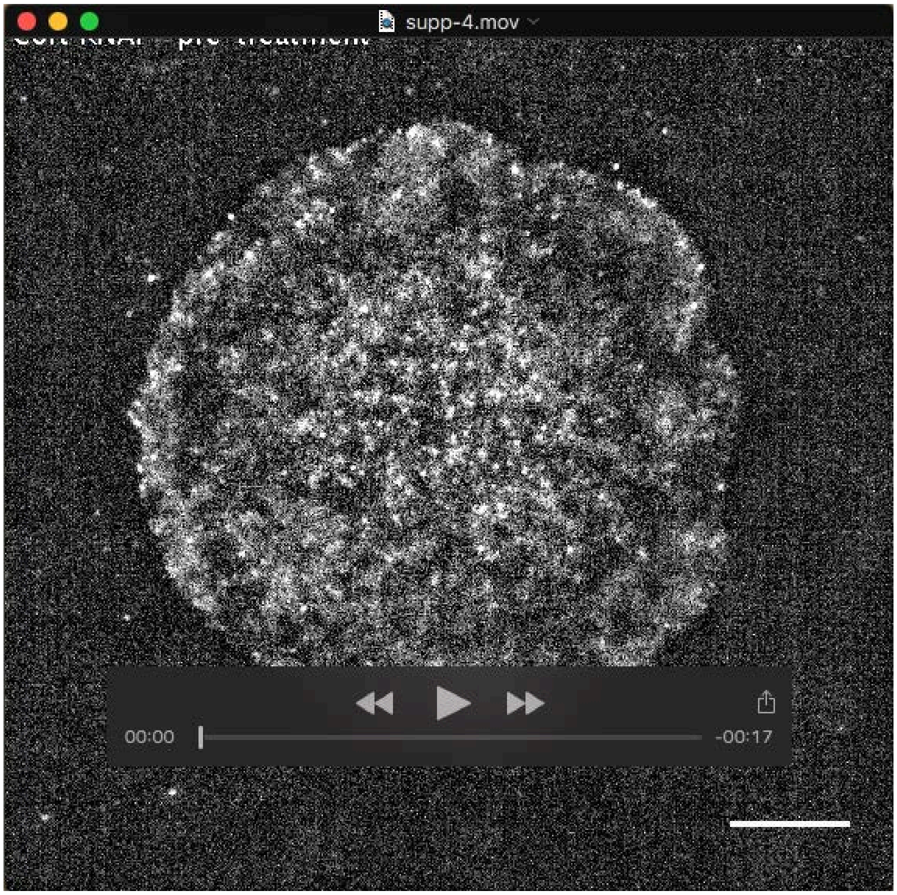

Movie 3. PARF of Naus following treatment with Cortactin RNAi. An S2R+ cell expressing EGFP-tagged Naus following Cortactin RNAi. After 40 seconds of imaging the cell was permeabilized with 25 μM digitonin. Image sequence was acquired at 2 second intervals.

Biology Open • Supplementary information

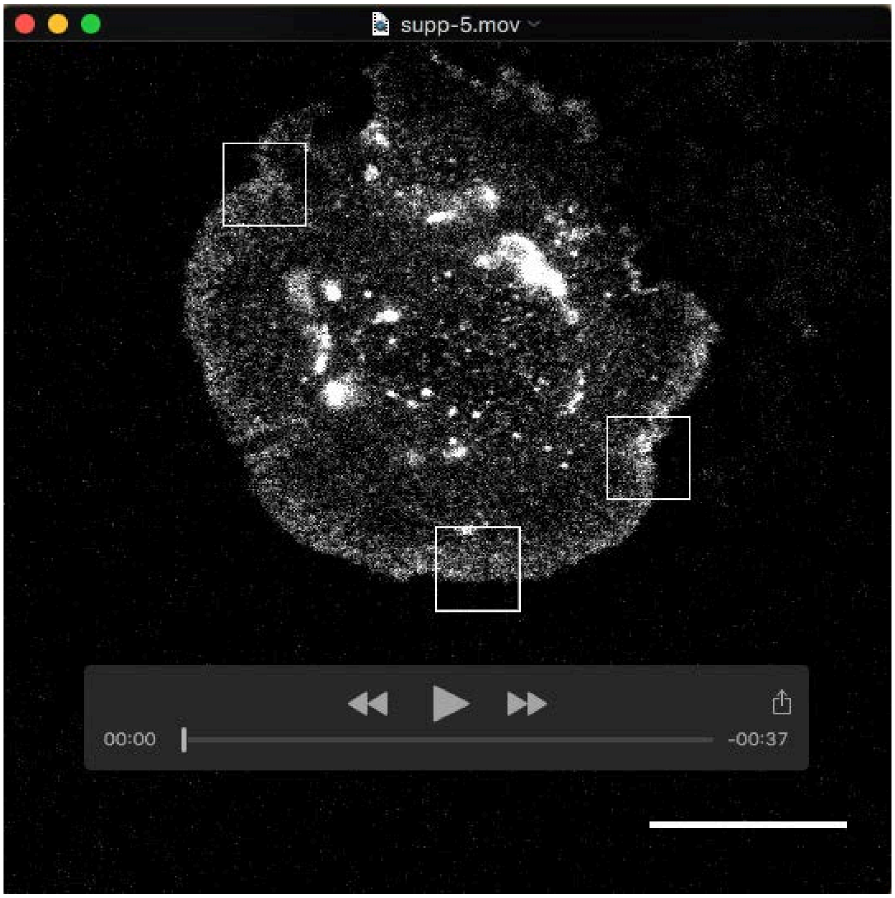

Movie 4. FRAP of Naus following treatment with control RNAi. An S2R+ cell expressing EGFP-tagged Naus following control RNAi. The cell was photobleached in the regions denoted by the white boxes. The cell was imaged by an LSM 880 confocal microscope at 2 second intervals.

Biology Open • Supplementary information

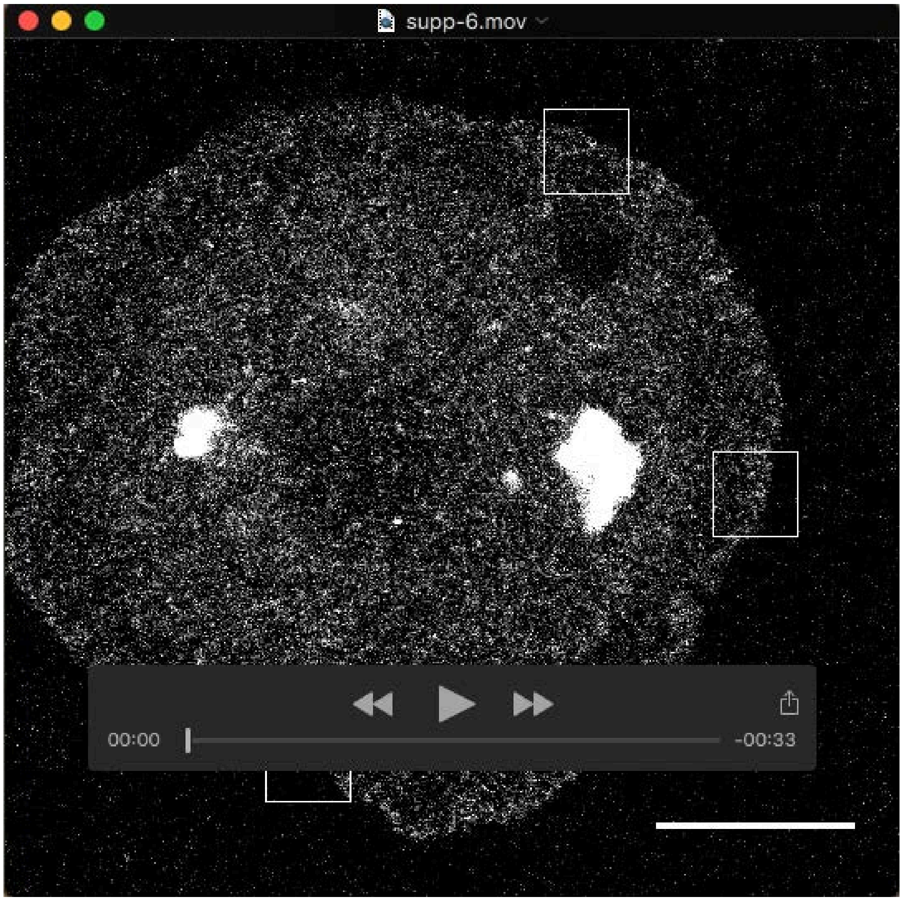

Movie 5. FRAP of Naus following treatment with Cortactin RNAi. An S2R+ cell expressing EGFP-tagged Naus following Cortactin RNAi. The cell was photobleached in the regions denoted by the white boxes. The cell was imaged by an LSM 880 confocal microscope at 2 second intervals.

Biology Open • Supplementary information

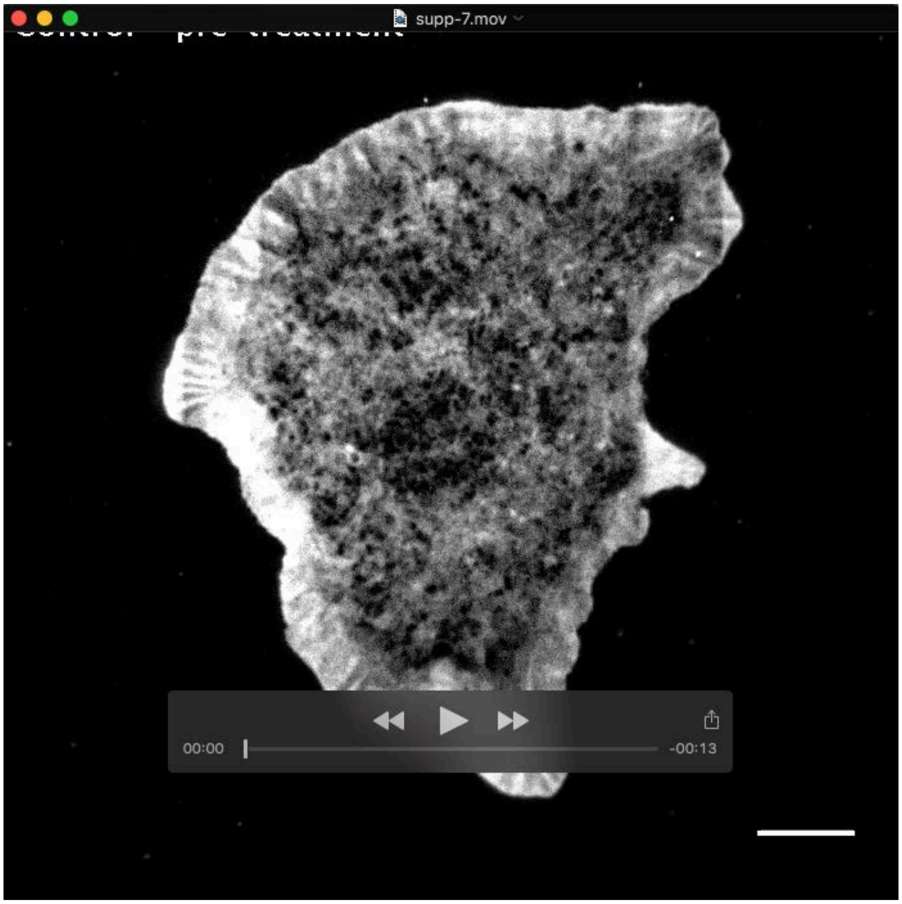

Movie 6. PARF of Cortactin following treatment with control RNAi. An S2R+ cell expressing mCherry-Cortactin following treatment with control RNAi. After 40 seconds of imaging the cell was permeabilized with 25 µM digitonin. Image sequence was acquired at 2 second intervals.

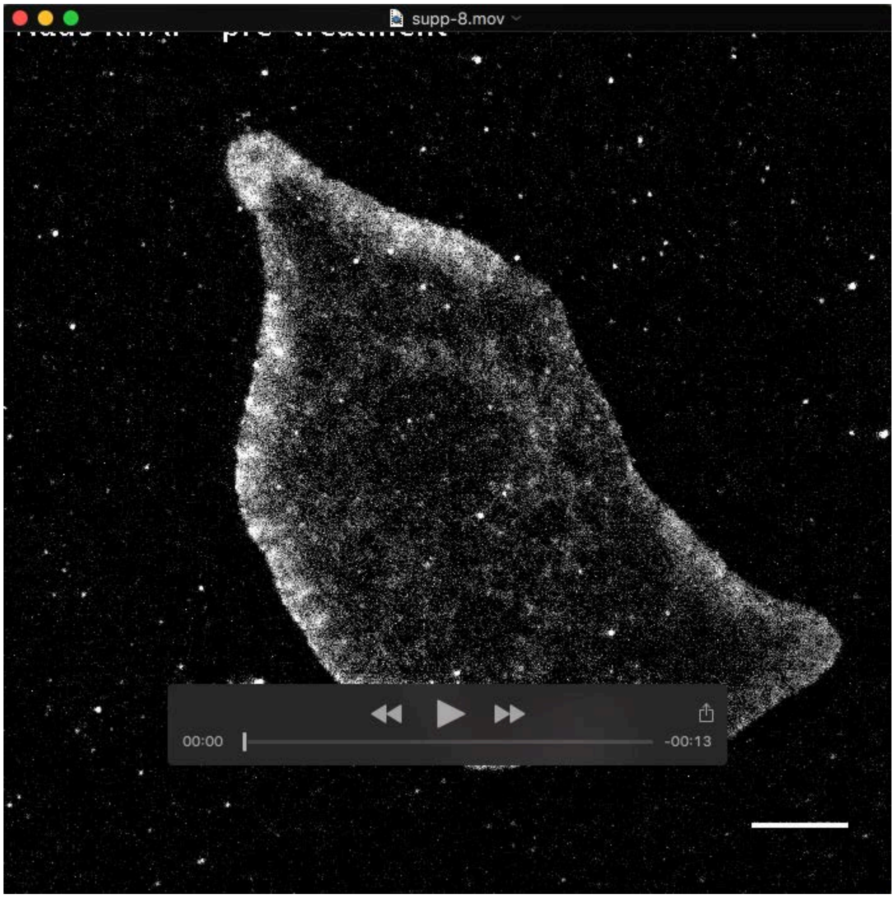

Movie 7. PARF of Cortactin following treatment with Naus RNAi. An S2R+ cell expressing mCherry-Cortactin following treatment with Naus RNAi. After 40 seconds of imaging the cell was permeabilized with 25 μM digitonin. Image sequence was acquired at 2 second intervals.

Biology Open • Supplementary information

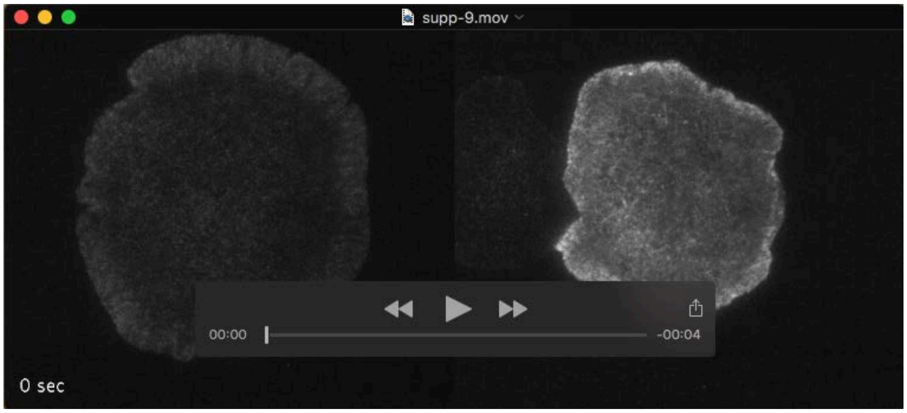

Movie 8. QFSM of EGFP-actin. Using a metallothionein promoter we titrated the addition of copper sulfate in order to generate actin speckles in S2R+ cells. When then imaged at 2 second the resulting actin dynamics following control (left) and Naus RNAi (right). Image analysis was carried out in Matlab.

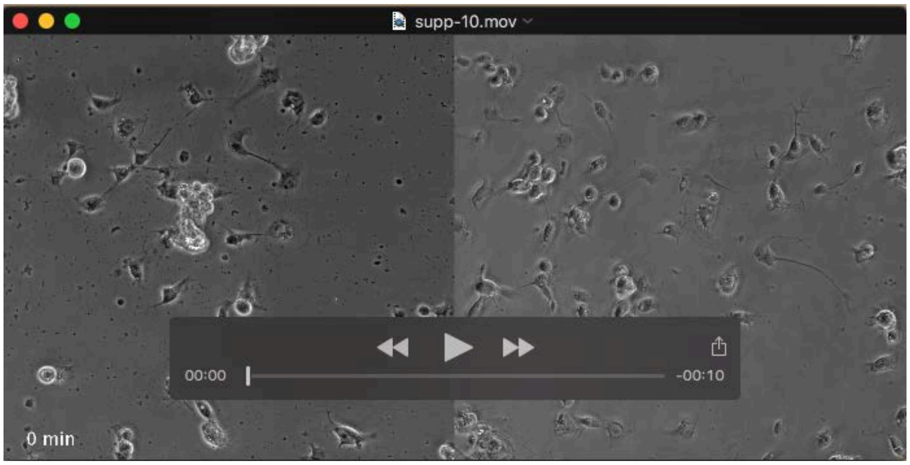

Movie 9. Random cell motility assay. D25 cells were treated with control or Naus RNAi for seven days and then were imaged by phase-contrast microscopy over a period of six hours. Image sequence was acquired at 5 minute intervals.
